# VEGFR1 and VEGFR2 Involvement in Extracellular Galectin-1- and Galectin-3-Induced Angiogenesis

**DOI:** 10.1371/journal.pone.0067029

**Published:** 2013-06-17

**Authors:** Nicky D'Haene, Sébastien Sauvage, Calliope Maris, Ivan Adanja, Marie Le Mercier, Christine Decaestecker, Linda Baum, Isabelle Salmon

**Affiliations:** 1 Department of Pathology, Erasme Hospital, Brussels, Belgium; 2 Laboratory of Image Synthesis and Analysis, Brussels School of Engineering/Ecole polytechnique de Bruxelles; Université Libre de Bruxelles (ULB), Brussels, Belgium; 3 DIAPATH – Center for Microscopy and Molecular Imaging (CMMI), ULB, Gosselies, Belgium; 4 Department of Pathology and Laboratory Medicine, UCLA School of Medicine, Los Angeles, California, United States of America; Katholieke Universiteit Leuven, Belgium

## Abstract

**Aim:**

Accumulating evidence suggests that extracellular galectin-1 and galectin-3 promote angiogenesis. Increased expression of galectin-1 and/or galectin-3 has been reported to be associated with tumour progression. Thus, it is critical to identify their influence on angiogenesis.

**Methods:**

We examined the individual and combined effects of galectin-1 and galectin-3 on endothelial cell (EC) growth and tube formation using two EC lines, EA.hy926 and HUVEC. The activation of vascular endothelial growth factor receptors (VEGFR1 and VEGFR2) was determined by ELISA and Western blots. We evaluated the VEGFR1 and VEGFR2 levels in endosomes by proximity ligation assay.

**Results:**

We observed different responses to exogenous galectins depending on the EC line. An enhanced effect on EA.hy926 cell growth and tube formation was observed when both galectins were added together. Focusing on this enhanced effect, we observed that together galectins induced the phosphorylation of both VEGFR1 and VEGFR2, whereas galectin-1 and −3 alone induced VEGFR2 phosphorylation only. In the same way, the addition of a blocking VEGFR1 antibody completely abolished the increase in tube formation induced by the combined addition of both galectins. In contrast, the addition of a blocking VEGFR2 antibody only partially inhibited this effect. Finally, the addition of both galectins induced a decrease in the VEGFR1 and VEGFR2 endocytic pools, with a significantly enhanced effect on the VEGFR1 endocytic pool. These results suggest that the combined action of galectin-1 and galectin-3 has an enhanced effect on angiogenesis via VEGFR1 activation, which could be related to a decrease in receptor endocytosis.

## Introduction

Angiogenesis, defined as the formation of new blood vessels from pre-existing vasculature, is one of the hallmarks of cancer described by Hanahan and Weinberg [1]. A significant amount of research on tumour angiogenesis has focused on vascular endothelial growth factor (VEGF) and methods to block its actions. Unfortunately, a significant number of patients do not respond to VEGF-targeted therapy [2]. This therapeutic failure may be at least partly explained by tumour cells most likely using multiple mechanisms to activate angiogenic signalling pathways.

Recently, extracellular galectin-1 and galectin-3 have been reported to promote angiogenesis [3], [4], [5], [6], [7], [8]. Galectins are animal lectins defined by their shared consensus amino acid sequences and their affinity for β-galactose-containing oligosaccharides Although most galectins bind preferentially to glycoproteins containing the ubiquitous disaccharide *N*-acetyl-lactosamine, individual galectins can also recognize different modifications to this minimum saccharide ligand and so demonstrate the fine specificity of galectins for specific ligands [9], [10], [11]. Thijssen et al. showed that tumour cells secrete galectin-1 to stimulate tumour angiogenesis [7]. Hsieh et al. showed that galectin-1 interacts with neuropilin-1 to activate VEGF receptor-2 (VEGFR2) signalling and modulates endothelial cell (EC) migration [3]. Extracellular galectin-3 stimulates angiogenesis *in vitro* and *in vivo* [6]. Recently, Markowska et al. demonstrated that galectin-3 modulates VEGF- and basic fibroblast growth factor (bFGF)-mediated angiogenesis by binding to αvβ3 integrin [5]. In addition, they found that galectin-3 can activate VEGFR2 by regulating receptor internalization [4].

Different studies have highlighted the diversity of ECs according to the organ or pathology (normal vs tumour) [12], [13], [14]. This heterogeneity was also observed regarding galectin-1 and galectin-3 expression in ECs. We and others have observed an overexpression of either galectin-1 or galectin-3 in tumour-associated ECs [8], [15], [16], [17], [18], [19]. In addition, the increased expression of galectin-1 and/or galectin-3 has been reported to be associated with tumour progression. To the best of our knowledge, few studies have examined the combined effects of galectin-1 and galectin-3 [20], [21], and no studies have examined their combined effects on angiogenesis. Thus, we decided to study the effects of exogenous galectin-1, galectin-3 and both galectins combined on angiogenesis-related events in two EC lines to assess the heterogeneity of ECs.

## Materials and Methods

### Reagents and cell culture

Human recombinant galectin-1 and galectin-3 were purchased from PeproTech (London, UK), blocking VEGFR1 antibody (Ab) was purchased from Abcam (Cambridge, UK) and blocking VEGFR2 Ab was purchased from R&D Systems (Abingdon, UK).

The human EA.hy926 EC line (ATCC number CRL-2922) was maintained in Dulbecco's modified Eagle's medium (DMEM) supplemented with 10% foetal bovine serum (FBS)_._ HUVEC cells (HUV-EC-C, ATCC, CRL-1730) were maintained in EGM2 bullet kit medium (Lonza, Verviers, Belgium). The EA.hy926 EC line is a hybridoma between HUVEC and the A549 lung carcinoma cell line [22]. The levels of endogenous galectin-1 and galectin-3 were similar between EA.hy926 and HUVEC cells ( Figure S1A), and galectin secretion was low (<8 ng/ml). The two cell lines were different in terms of VEGFR expression, i.e., EA.hy926 cells were characterised by higher VEGFR1 and lower VEGFR2 expression compared to HUVECs (Figure S1A).

### Cell Growth Assay

EA.hy926 (3×10^3^) or HUVEC (2×10^3^) cells were seeded into 96-well plates in complete growth medium. For EA.hy926 cells, after 24 h, cells were starved for an additional 24 h in serum-free medium (SFM). Adherent cells were then pulsed with galectin-1, galectin-3 or both at different concentrations in SFM. After 24 h, the MTT assay was performed as previously described [23]. For HUVEC cells, after 24 h, adherent cells were pulsed with galectin-1, galectin-3 or both at different concentrations in serum-free EBM2. After 48 h, the MTT assay was performed as previously described [23]. Each condition contained six replicates.

### *In vitro* tube formation

Unpolymerised growth factor-reduced matrigel (8.7 mg/ml; B&D Biosciences, Bedford, MA) was placed in µ-slide angiogenesis (Ibidi, Beloeil, Belgium) (10 µl/well) and allowed to polymerise for 1 h at 37°C. We first performed a kinetic study of tube formation with different cell concentrations. This study revealed that tube formation was maximal after 6 h at the concentration of 3×10^3^ cells/well for HUVECs, and after 22 h at the concentration of 12×10^3^ cells/well for EA.hy926 cells (data not shown). EA.hy926 cells (12×10^3^ cells/well) or HUVECs (3×10^3^ cells/well) were suspended in complete medium with or without different concentrations of galectins in the presence or absence of 5 µg/ml mouse monoclonal blocking VEGFR1 Ab or 50 ng/ml mouse monoclonal blocking VEGFR2 Ab or in presence of control IgG (Dako, Glostrup, Denmark) at the same concentration that the blocking Ab under analysis. The cells were seeded on top of the matrigel layer and incubated at 37°C. After 6 h for HUVECs and 22 h for EA.hy926 cells, the wells were photographed using an inverted phase-contrast microscope (Olympus, Aartselaar, Belgium). HUVECs and EA.hy926 cells formed capillary-like networks with different tube morphology, as observed by Shtivelband et al. [24] (Figure S1B). Tube formation was quantified by measuring the total length of the tube network and the number of branching points under 2× magnification using ImageJ software (NIH, Bethesda). Tube

### Enzyme-Linked Immunosorbent Assays

The levels of phosphorylated VEGFR1, VEGFR2, extracellular signal-regulated kinase (ERK)1/2, heat-shock protein 27 (Hsp27), Src, protein kinase B (Akt) and focal adhesion kinase (FAK) were examined using human-specific phospho ELISAs (R&D Systems) (Materials and Methods S1). Each condition was evaluated in two independent experiments that were performed in triplicate.

### Western blots

EA.hy926 lysates were analyzed by Western blots, as previously detailed [23]. Total and phosphorylated protein expression levels were evidenced by means of specific anti-human Abs against VEGFR1 (Abcam, 1/1000), phospho-VEGFR1 (R&Dsytems, 1 µg/ml), VEGFR2 (Cell Signaling, Beverly, MA, 1/1000), phospho-VEGFR2 (Cell Signaling, 1/500), ERK 1/2(R&Dsytems, 0.5 µg/ml), phospho-ERK 1/2 (R&Dsytems, 0.1 µg/ml), Hsp27 (R&Dsytems, 0.1 µg/ml), phospho-Hsp27(R&Dsytems, 0.1 µg/ml), FAK (R&Dsytems, 1 µg/ml), phospho-FAK (R&Dsytems, 2 µg/ml), Src (R&Dsytems, 0.1 µg/ml), phospho-Src (R&Dsytem, 1 µg/ml), Akt (R&Dsytems, 1 µg/ml) and phospho-Akt (R&Dsytems, 1 µg/ml). Evaluation of total proteins was performed on the membranes corresponding to their phosphorylated forms after stripping using Restore Western Blot Stripping Buffer (Thermo Scientific) according to the manufactorer's protocol. The monoclonal anti-tubulin Ab (Abcam,1/5000) was used as loading control. Quantification of Western blots was done using ImageJ software by integrating the band intensity followed by normalization with regard to tubulin and expressed as a fold change compared with the control (no galectin addition).

### Proximity ligation assay

We used the Duolink in situ PLA kit from Olink Bioscience (Olink Bioscience, Uppsala, Sweden) to detect colocalisation between VEGFR1 or VEGFR2 and early endosome antigen-1 (EEA1) according to the manufacturer's instructions (Materials and Methods S1). The PLA signal/cell was determined with image analysis software developed by the Laboratory of Image Synthesis and Analysis (ULB, Brussels, Belgium) (Materials and Methods S1). Each condition was evaluated in two independent experiments.

### Statistical analyses

The non-parametric Kruskal-Wallis test was used to compare multiple independent groups of numerical data. If the test was significant, post-hoc tests were applied using either the standard Dunn procedure to compare all group pairs or its adaptation to compare each experimental condition to the control, avoiding multiple comparison effects (as detailed in Zar [25]).

To evaluate whether the combined effect induced by the two galectins was additive or synergistic (the latter being defined as a total effect greater than the sum of the individual effects), we used the adjusted rank transform test described by Leys et al [26].

All statistical analyses were performed using Statistica (Statsoft, Tulsa, OK, USA).

## Results

### Modulation of cell growth by exogenous galectins

**Figure 1:**
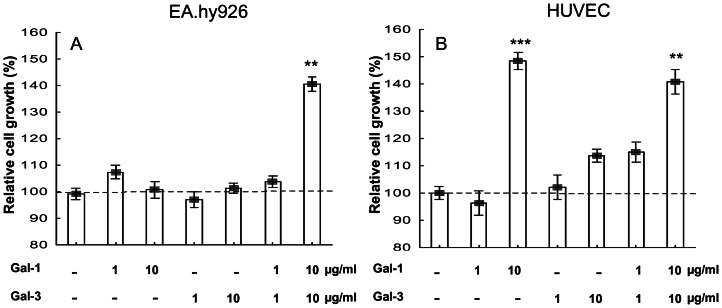
Modulation of cell growth by exogenous galectins. The effects of exogenous galectins on the growth of EA.hy926 (A) and HUVEC (B) cells were evaluated by the MTT assay. (A) The MTT conversion in EA.hy926 cells was measured one day after galectin stimulation at the indicated concentrations. (B) The MTT conversion in HUVECs was measured two days after galectin stimulation at the indicated concentrations. Each condition was tested with six replicates. The data (mean +/− SEM) are shown as relative values compared with the control (no galectin addition), and significant differences are indicated (* p<0.05, ** p<0.01 and *** p<0.001).

We observed different responses to exogenous galectins depending on the cell line. In EA.hy926 cells, we observed no significant effect of galectin-1 or galectin-3 (Figure 1A). In contrast, the addition of both galectins together at 10 µg/ml each increased cell growth by 43% (p<0.01).

Galectin-1 alone significantly increased HUVEC growth at 48 h by 47% (p<0.001; Figure 1B). A slight but not statistically significant increase (12%) was also observed for galectin-3 alone (10 µg/ml). The addition of both galectins together (10 µg/ml each) to the culture medium increased cell growth to a similar level as galectin-1 alone (Figure 1B).

### Modulation of tube formation by exogenous galectins

**Figure 2:**
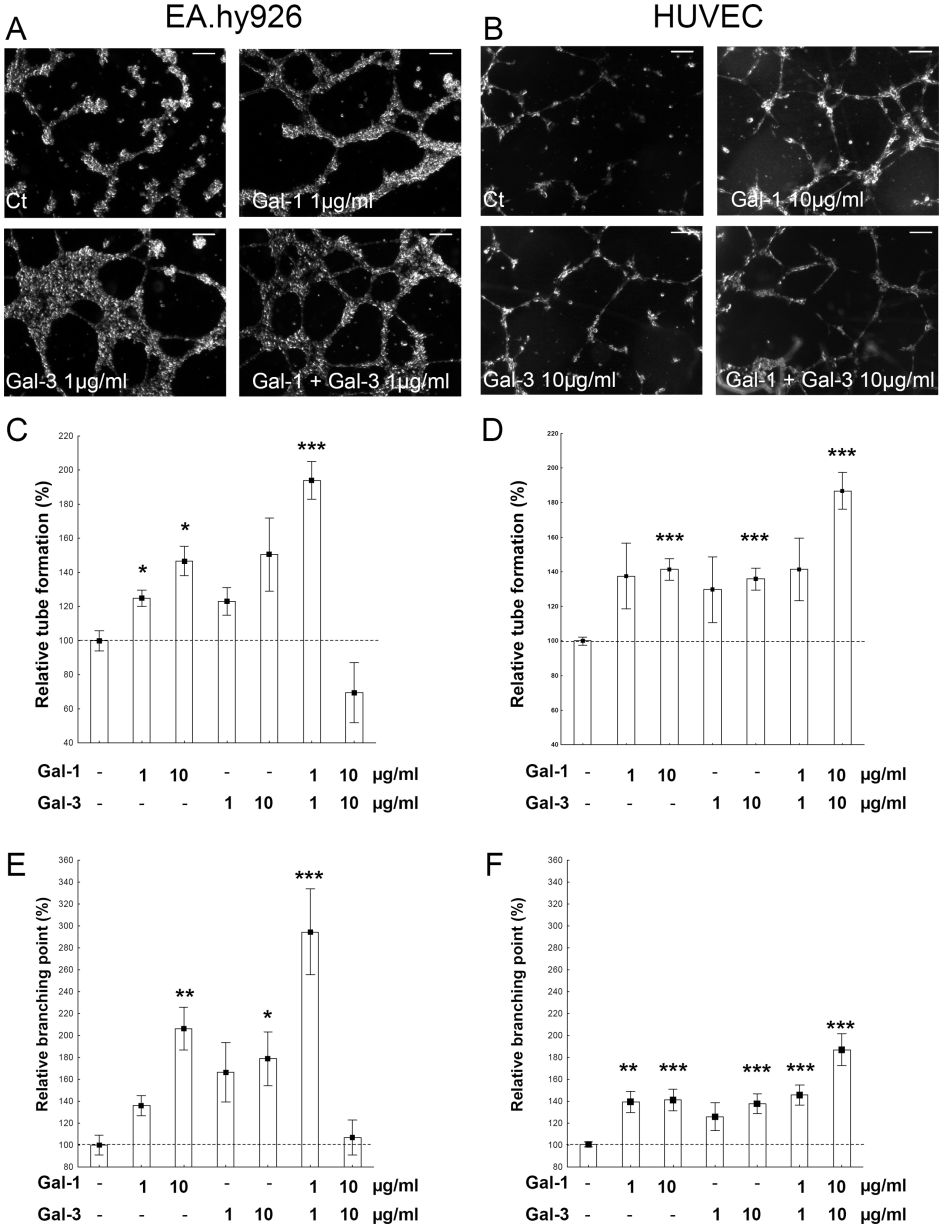
Modulation of tube formation by exogenous galectins. EA.hy926 (A, C, E) and HUVEC (B, D, F) cells were suspended in complete medium in the presence or absence of galectins at the indicated concentrations and seeded on top of matrigel layers. Representative images obtained at 22 h for EA.hy926 (A) and 6 h for HUVEC (B) are shown. Tube formation was quantified by measuring the total length of the tube network (C, D) or by counting branching point (E, F) in EA.hy926 cells (C, E) and HUVECs (D, F). The data (mean +/− SEM) are shown as relative values compared with the control (no galectin addition), and significant differences are indicated (* p<0.05, ** p<0.01 and *** p<0.001). Scale bar: 300 µm.

In both EA.hy926 cells and HUVECs, the addition of galectin-1 or galectin-3 alone stimulated tube formation (Figure 2). Regarding EA.hy926 cells, the addition of both galectins together at 1 µg/ml each induced a significant and synergistic effect on the total tube length (average tube length increase of 25%, 23% and 94% in response to galectin-1, galectin-3 and galectin-1+ galectin-3, respectively) (Figures 2A, C), paralleled by a significant effect on branching point numbers (average branching point number increase of 36%, 67% and 195% in response to galectin-1, galectin-3 and galectin-1+ galectin-3, respectively) (Figure 2E). This effect was not statistically detected as synergistic, probably because of a larger heterogeneity in these data compared with the tube length. The addition of both galectins at 10 µg/ml each had an antagonistic effect on tube length and branching points (average tube length increase of 47% and 50% and average branching point number increase of 106% and 79% in response to galectin-1 and galectin-3, respectively, in contrast to an average tube length decrease of 31% and an average branching point number increase of only 7% in response to galectin-1+ galectin-3) (Figures 2C, E).

In contrast, in HUVECs, an additive effect on tube length and branching point number was induced by the addition of both galectins at 10 µg/ml each (average tube length increase of 41%, 36% and 87% and average branching point number increase of 41%, 38% and 87% in response to galectin-1, galectin-3 and galectin-1+ galectin-3, respectively) (Figures 2B, D, F).

### Galectin-induced tube formation is related to VEGFR activation

**Figure 3:**
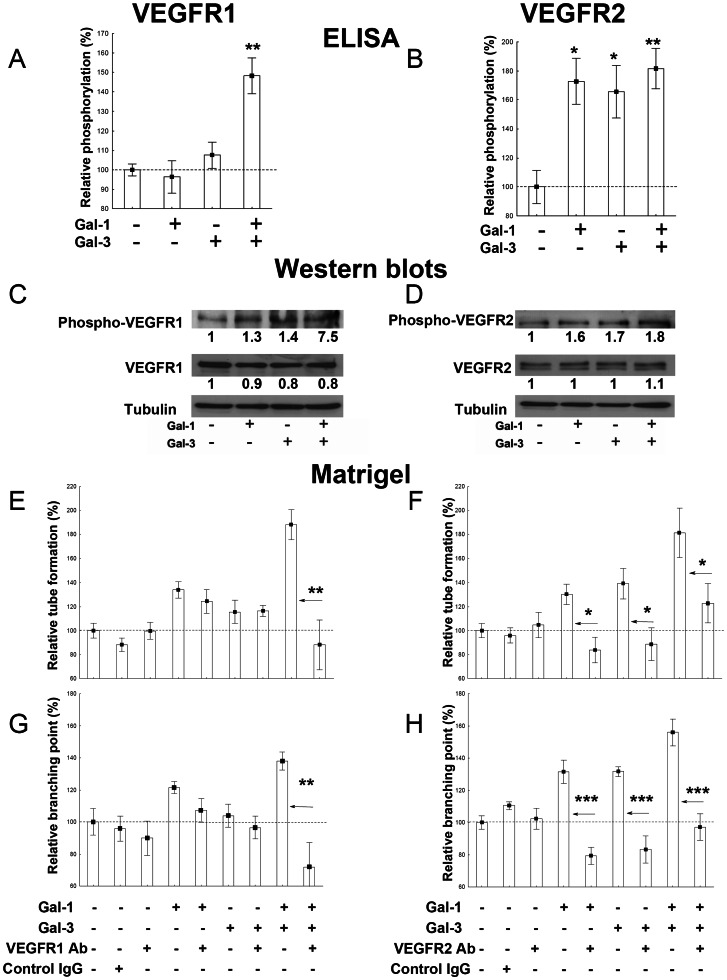
Effects of exogenous galectins on VEGFR activation and involvement of VEGFRs in galectin-induced tube formation. (A–D) Determination of VEGFR1 (A, C) and VEGFR2 (B, D) phosphorylation levels following a 5-min stimulation of EA.hy926 cells with galectin-1, galectin-3 or both galectins (1 µg/ml each) by ELISA (A, B) and Western blots (C, D). For ELISAs, the data (mean +/− SEM) are shown as relative values compared with the control (no galectin addition), and significant differences are indicated (* p<0.05, ** p<0.01 and *** p<0.001). Quantification of Western blots was done using ImageJ (see Materials and Methods). (E–H) EA.hy926 cells were suspended in complete medium in the presence or absence of galectins (1 µg/ml each) and blocking VEGFR1 Ab (5 µg/ml) or control IgG (5 µg/ml) (E, G) or blocking VEGFR2 Ab (50 ng/ml) or control IgG (50 ng/ml) (F, H) and seeded on top of matrigel layers. Tube formation was quantified by measuring the total length of the tube network (E–F) or counting branching points (G–H). The data (mean +/− SEM) are shown as relative values compared with the control (without the addition of galectins or an inhibitor). Significant differences are indicated on horizontal arrows (the same galectin-related conditions were compared in the absence or presence of a blocking Ab using the Mann-Whitney test. * p<0.05, ** p<0.01 and *** p<0.001).

Based on the EC response to galectins, we next investigated the enhanced effect induced by galectin-1 and galectin-3 added together at 1 µg/ml each on EA.hy926 cells. We determined the pathways underlying this galectin-induced stimulation. Previous studies have shown that galectin-1 and galectin-3 can activate VEGFR2 [3], [4]. However, we did not find any data in the literature (to the best of our knowledge) related to the galectin-induced activation of VEGFR1. Therefore, we analysed the expression and phosphorylation levels (by ELISA and western blotting) of VEGFR2 and VEGFR1 in EA.hy926 cells after galectin stimulation. The addition of galectin-1, galectin-3 or both galectins together had no effect on VEGFR1 or VEGFR2 protein expression (Figures 3C–D). As shown in Figs. 3A–D, galectin-1 and galectin-3 alone induced VEGFR2 phosphorylation without VEGFR1 phosphorylation. In contrast, the addition of both galectins together induced VEGFR1 and VEGFR2 phosphorylation. VEGFR2 activation was inhibited by lactose but not sucrose, indicating that the effect is due to glycan binding by galectins (Figure S2).

Next, we examined whether the galectin-induced activation of VEGFRs was involved in galectin-induced tube formation. For this purpose, we added either blocking VEGFR1 Ab or blocking VEGFR2 Ab to EA.hy926 cells plated on matrigel in the presence of galectins. In agreement with our observations of VEGFR phosphorylation, the increase in tube formation induced by galectin-1 and galectin-3 alone was abolished by the addition of blocking VEGFR2 Ab (p = 0.02 and 0.01, respectively regarding total tube length evaluation and p<0.001 for both regarding branching point evaluation; Figures 3F, H), whereas blocking VEGFR1 Ab did not affect the effects induced by each galectin alone (Figures 3E, G). Furthermore, the addition of blocking VEGFR1 Ab completely abolished the enhanced tube formation observed when both galectins were added together (p = 0.002 regarding tube length evaluation and p = 0.008 regarding branching points evaluation; Figures 3E, G). In contrast, the addition of blocking VEGFR2 Ab only partially inhibited the enhanced effect observed regarding tube length (p = 0.02 regarding tube length evaluation and p = 0.0007 regarding branching point evaluation; Figures 3F, H).

**Figure 4:**
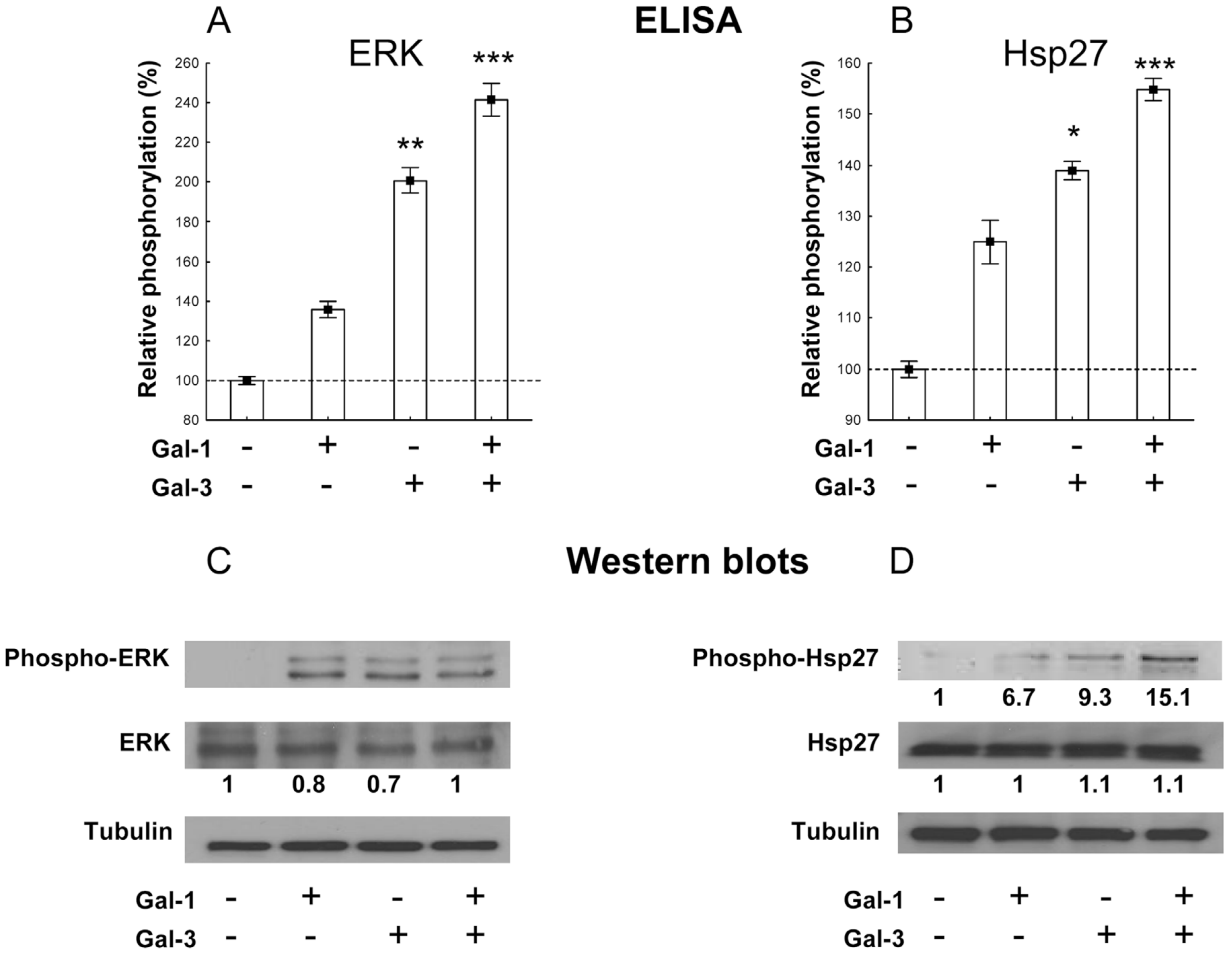
Galectin-induced activation of ERK1/2 and Hsp27. Determination of ERK1/2 (A, C) and Hsp27 (B, D) phosphorylation levels following a 10-min stimulation of EA.hy926 cells with galectin-1, galectin-3 or both galectins (1 µg/ml each), by ELISA (A, B) and Western blots (C, D). For ELISAs, the data (mean +/− SEM) are shown as relative values compared with the control (no galectin addition), and significant differences are indicated (* p<0.05, ** p<0.01 and *** p<0.001). Quantification of Western blots was done using ImageJ (see Materials and Methods).

We then examined the signalling pathways that might underlie galectin-induced VEGFR activation. Galectin-1 and galectin-3 have been shown to activate ERK1/2 and FAK, respectively [3], [5], and the major pathways of VEGFR2 signal transduction include ERK1/2, Akt, Src, FAK and Hsp27 activation [27]. Therefore, using ELISA and Western blots, we examined whether the galectins added alone or together would differentially activate these pathways. The addition of galectin-1, galectin-3 or both galectins together had no effect on Akt, Src or FAK protein expression (evaluated by Western blots; data not shown). No phosphorylation of Akt, Src and FAK was observed (data not shown). The addition of galectins induced ERK and Hsp27 phosphorylation evaluated by ELISA or Western blots (Figure 4). When both galectins were added together, additive effects were observed for ERK phosphorylation (average increase of 36%, 101% and 142% evaluated by ELISA in response to galectin-1, galectin-3 and galectin-1+ galectin-3, respectively –Western blot quantification was not possible due to absence of phosphorylation in the control condition) and HSp27 (average increase of 25%, 39% and 55% evaluated by ELISA and fold change of 6.7, 9.3 and 15.1 evaluated by Western blots in response to galectin-1, galectin-3 and galectin-1+ galectin-3, respectively).

### Modulation of VEGFR endocytosis by exogenous galectins

**Figure 5:**
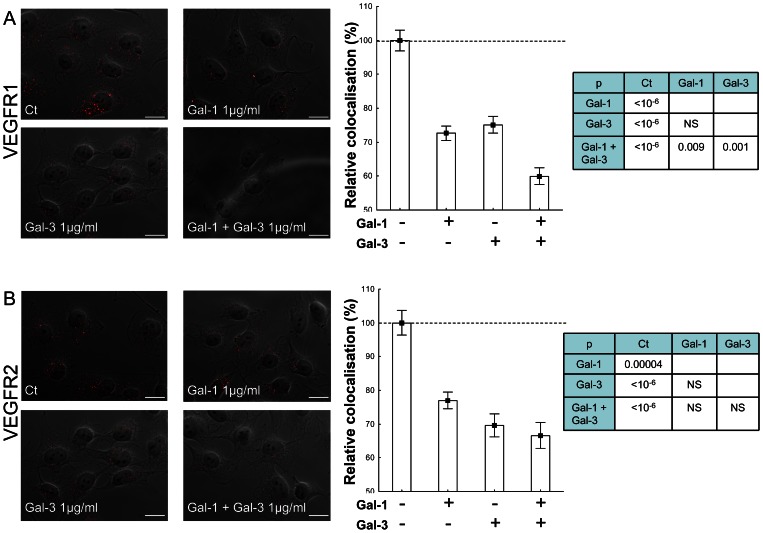
Modulation of VEGFR endocytosis by exogenous galectins in EA. hy926 cells. The effects of exogenous galectins (1 µg/ml each) were evaluated by analysing the colocalisation between each receptor and EEA1 using the proximity ligation assay and an image analysis tool. Representative images of z-stacks of 7 fluorescent micrographs projected into a single phase-contrast image (original magnification: ×60) are shown. Signal/cell values are shown as relative values (mean +/− SEM) compared with the control (no galectin addition). The tables show the significance levels obtained by applying the standard Dunn procedure (post-hoc test) to compare all the pairs of experimental conditions, in order to avoid multiple comparison effects (NS  =  not significant: p>0.05). Scale bar: 20 µm.

Because previous studies have highlighted the role of galectin lattices in the control of receptor turnover and endocytosis [28] and shown that galectin-3 retains VEGFR2 on the plasma membrane [4], we analysed whether the endocytosis of VEGFRs could be modulated by galectins. Previous studies have shown that endocytic pool of VEGFR2 is related to early endosomes [29], [30]. Thus, the VEGFR1 and VEGFR2 levels in early endosomes were evaluated by studying the colocalisation of early endosomal antigen 1 (EEA1) and VEGFR1 or VEGFR2 using the proximity ligation assay (Figure 5). First, control conditions with or without BSA were studied by proximity ligation assays; the results showed no statistically significant difference between the two conditions (data not shown). The addition of galectin-1 or galectin-3 to the culture medium was followed by a significant decrease in the VEGFR1 and VEGFR2 endocytic pool (average decrease between 23 and 30%). The addition of both galectins together decreased the VEGFR2 endocytic pool to a similar level as galectin alone (average decrease of 33%; Figure 5B). In contrast, the decrease in the VEGFR1 endocytic pool was significantly more pronounced when both galectins were added (average decrease of 40%, p = 0.009 and 0.001 in comparison to galectin-1 and galectin-3 alone, respectively; Figure 5A). These findings indicate that galectin-1 and galectin-3 reduce VEGFR1 and VEGFR2 internalisation, which is consistent with the galectin lattice retaining these receptors on the plasma membrane [28].

Taken together, these results suggest that galectin-1 and galectin-3 have an enhanced effect on EA.hy926 tube formation via VEGFR1 activation, which could be related to a decrease in receptor endocytosis.

## Discussion

In agreement with previous studies [3], [4], [5], [6], [7], the current study shows that galectin-1 and galectin-3 can differently stimulate angiogenesis. The major finding of the current study is that when added together, exogenous galectin-1 and galectin-3 had enhanced effects on angiogenesis related-events in EA.hy926 cells (with a biphasic effect on tube formation) compared to the reduced effects induced by each galectin separately. The EA.hy926 cell response to galectin-1 or galectin-3 stimulation was characterised by VEGFR2 activation, as previously described [3], [4]. When both galectins were added together, we observed both VEGFR2 and VEGFR1 phosphorylation. We believe that the enhanced effect observed when both galectins were combined could be related to VEGFR1 activation because the galectins separately did not induce VEGFR1 phosphorylation. The precise function of VEGFR1 is still a subject of debate. The weak tyrosine kinase activity of VEGFR1 and its high affinity for VEGF suggest a model in which VEGFR1 acts as a negative modulator of VEGF-mediated angiogenesis [27]. However, other reports indicate that VEGFR1 may instead promote angiogenesis under pathological conditions [14], [31]–[33]. Indeed, these studies evidenced that the activation of VEGFR1 results in the amplification of angiogenesis mediated by VEGFR2, as we observed in the present study [14], [32], [33]. In the same manner, the addition of blocking VEGFR1 antibody completely abolished the enhanced stimulation of tube formation when both galectins were added together. In contrast, the addition of blocking VEGFR2 antibody only partially inhibited this enhanced effect (Figs. 3C–D).

These results suggest that galectin-1 and −3 are angiogenic molecules that activate components of VEGF signalling pathways, suggesting that these galectins could promote such pathways. It would thus be interesting to study the possible interactions between these galectins and VEGF. In addition, because VEGFR1 is activated in EA.hy926 cells by the combined effects of these two galectins, it would also be informative to evaluate their effects on the secretion of VEGFR1 ligands, such as placental growth factor (PlGF) and VEGF-B. Recently, Markowska et al. highlighted the role of galectin-3 in angiogenic intracellular signal transmission mediated by VEGF and bFGF [5].

One mechanism through which the two galectins might mediate VEGFR activation is by increasing the density of these receptors on the cell surface, making them accessible to low levels of endogenous VEGF. Consistent with this model, we observed that galectin-1 and galectin-3 decreased the levels of internalised VEGFR1 and VEGFR2 and that the presence of both galectins enhanced the decrease in the internalised VEGFR1 pool. This latter observation reinforces our hypothesis that VEGFR1 is involved in enhanced angiogenesis induced by the combined action of galectin-1 and galectin-3. Our findings are also in agreement with the role of galectin in lattice formation, as recent literature has shown that members of the galectin family (including galectin-1 and galectin-3) regulate the plasma membrane residency of glycoproteins, including growth factor receptors [28].

Signaling pathways downstream of VEGF receptors and activated following the addition of galectins involve the MAP kinase pathway (ERK) and Hsp27. Activation of ERK may be involved in the proliferative effect induced by galectins while Hsp27 in cell migration and tube formation [27]. Our results are in agreement with those of Hsieh et al. showing that galectin-1 activates ERK1/2 [3]. Galectin-3 has been shown to trigger FAK activation in HUVEC cells [5]. No phosphorylation of FAK was observed in the present study. This difference can be explained by methodological differences. Indeed, Markowska et al. [5] stimulated the cells with higher concentrations (10 µg/ml) of galectin-3 compared to our experiments (1 μg/ml).

The two cell lines used in the current study (HUVEC and EA.hy926) showed different responses to galectins in terms of cell growth and tube formation, highlighting the heterogeneity of ECs and EC lines. This cell line-dependent response to galectins could be because the two cell lines are different in terms of VEGFR expression. Indeed, EA.hy926 cells are characterised by higher VEGFR1 and lower VEGFR2 expression compared to HUVECs (Figure S1). Variations in VEGFR expression have already been observed for ECs during hypoxia or VEGF stimulation, which stimulates VEGFR1 expression but decreases VEGFR2 levels in ECs [34], [35]. Together with the study of Zhang et al. [36], which demonstrated that VEGFR1 expression is increased in tumour-associated ECs of head and neck carcinomas, these data emphasise the importance of evaluating VEGFR expression in human tissues to optimize targeted therapies. The evaluation of VEGFR1 and VEGFR2 expression in a series of human normal and tumour tissues is currently underway in our laboratory.

The results of the current study lead us to hypothesise that the EC response to extracellular galectins could be regulated by the environment. In ECs characterised by high VEGFR2 and low VEGFR1 expression, extracellular galectin-1 and galectin-3 induced angiogenesis via activation of the VEGFR2 signalling pathway, with an additive effect in the presence of both galectins. In ECs characterised by low VEGFR2 and high VEGFR1 expression, extracellular galectin-1 and galectin-3 separately induced angiogenesis via activation of the VEGFR2 signalling pathway, whereas a synergistic effect was observed in the presence of both galectins via activation of the VEGFR1 signalling pathway.

## Supporting Information

Figure S1 **Characterisation of EA.hy926 and HUVEC cell lines.** (A) Characterisation of VEGFR and galectin expression in HUVEC and EA.hy926 lysates by western blotting. Protein expression was examined using specific anti-human Abs against galectin-1 (1∶1000; PeproTech), galectin-3 (1∶1000; Novocastra, Newcastle, UK), VEGFR1 (1∶1000; Abcam) and VEGFR2 (1∶1000; Cell Signaling, Beverly, MA). Monoclonal anti-tubulin Ab (1∶5000; Abcam) served as a loading control. (B) When plated on matrigel, HUVECs and EA.hy926 cells formed capillary-like networks with different tube morphology. HUVEC tubes were thin and lined with a single cell layer, but EA.hy926 tubes were more complex, with larger diameters that were formed by clumps of cells. HUVEC tubes were characterised by dichotomous branching, but EA.hy926 tubes displayed heterogeneous branching with uneven diameters. The formation of capillary-like networks was slower for EA.hy926 cells (22 h) compared with HUVECs (6 h).
(TIF)

Figure S2 **The VEGFR2 activation induced by galectin-1 and galectin-3 was inhibited by lactose but not sucrose, indicating that the effect is due to glycan binding by galectins.** VEGFR2 phosphorylation levels in EA.hy926 cells following a 5-min stimulation with both galectins (1 µg/ml) in the absence or presence of lactose or sucrose (50 mmol/l). The data are presented as the mean +/− SEM (* p<0.05).
(TIF)

Materials and Methods S1 (DOC)
