# Correction: VEGFR1 and VEGFR2 Involvement in Extracellular Galectin-1- and Galectin-3-Induced Angiogenesis

**DOI:** 10.1371/journal.pone.0295736

**Published:** 2023-12-07

**Authors:** Nicky D’Haene, Sébastien Sauvage, Calliope Maris, Ivan Adanja, Marie Le Mercier, Christine Decaestecker, Linda Baum, Isabelle Salmon

The VEGFR2 Gal-3 1 µg/ml panel in Fig 5B [1] is incorrect as it is an inadvertent duplication of the VEGFR1 Gal-3 1μg/ml panel in Fig 5A. The corrected Fig 5 is provided with this notice, and original data underlying this figure are provided in S1 File.

The Western blot panels in Fig 4C and 4D showing tubulin, total and phosphorylated protein expression were performed on the same gel, and the uncropped images and data tables underlying these results are provided in S2 File.

In the article [1], the following results are described but the data were not shown. The data supporting these results are provided with this notice in the indicated Supporting Information files:

- Tube formation was maximal after 22 h at the concentration of 12×10^3^ cells/well in EA.hy926 cells (S3 File).
- Akt and Src protein expression and phosphorylation were evaluated by Western blot. The article stated that no phosphorylation was observed; however, this should be corrected to state that no effect of galectin on phosphorylation of Src was observed (S4 File).

The first author stated that tube formation results for HUVEC cells, Western blots underlying FAK expression, and the data proximity ligation assays in control conditions with or without BSA are no longer available. However, the data underlying the rest of the results in this article [1] are available on request from the first author, Nicky D’Haene at nicky.dhaene@hubruxelles.be.

The authors apologize for the inadvertent image duplication in Fig 5.

**Fig 5:**
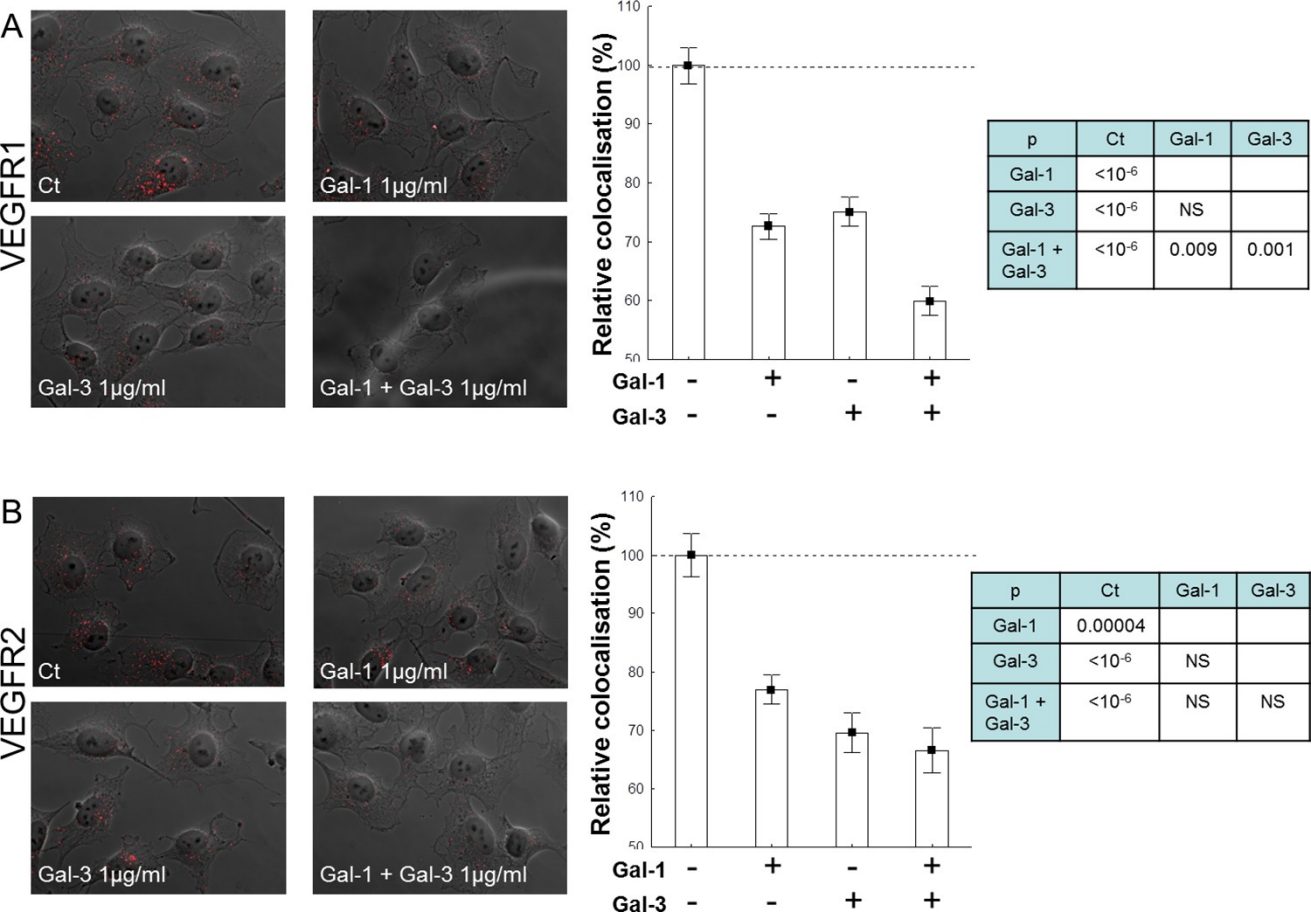
Modulation of VEGFR endocytosis by exogenous galectins in EA.hy926 cells. The effects of exogenous galectins (1 µg/ml each) were evaluated by analysing the colocalisation between each receptor and EEA1 using the proximity ligation assay and an image analysis tool. Representative images of z-stacks of 7 fluorescent micrographs projected into a single phase-contrast image (original magnification: ×60) are shown. Signal/cell values are shown as relative values (mean +/− SEM) compared with the control (no galectin addition). The tables show the significance levels obtained by applying the standard Dunn procedure (post-hoc test) to compare all the pairs of experimental conditions, in order to avoid multiple comparison effects (NS   =   not significant: p>0.05). Scale bar: 20 µm.

## Supporting information

S1 File Original data underlying Fig 5. (ZIP)

S2 File Original data underlying Fig 4C and 4D. (ZIP)

S3 File Tube formation in EA.hy926 cells. Tube formation was maximal after 22 h at the concentration of 12×10^3^ cells/well for EA.hy926 cells.
(ZIP)

S4 File Western blots for Akt and Src protein expression and phosphorylation. The addition of galectin-1, galectin-3 or both galectins together had no effect on Akt or Src protein expression or Src phosphorylation evaluated by Western blot.
(PPTX)

## References

[1] D’Haene N, Sauvage S, Maris C, Adanja I, Le Mercier M, Decaestecker C, et al. (2013) VEGFR1 and VEGFR2 Involvement in Extracellular Galectin-1- and Galectin-3-Induced Angiogenesis. PLoS ONE 8(6): e67029. 10.1371/journal.pone.0067029 23799140 PMC3684579

