# Supplementary figures and images for: VEGFR1 and VEGFR2 Involvement in Extracellular Galectin-1- and Galectin-3-Induced Angiogenesis
Source: PLoS One. 2013 Jun 17;8(6):e67029. doi: 10.1371/journal.pone.0067029 (PMC3684579; doi:10.1371/journal.pone.0067029)

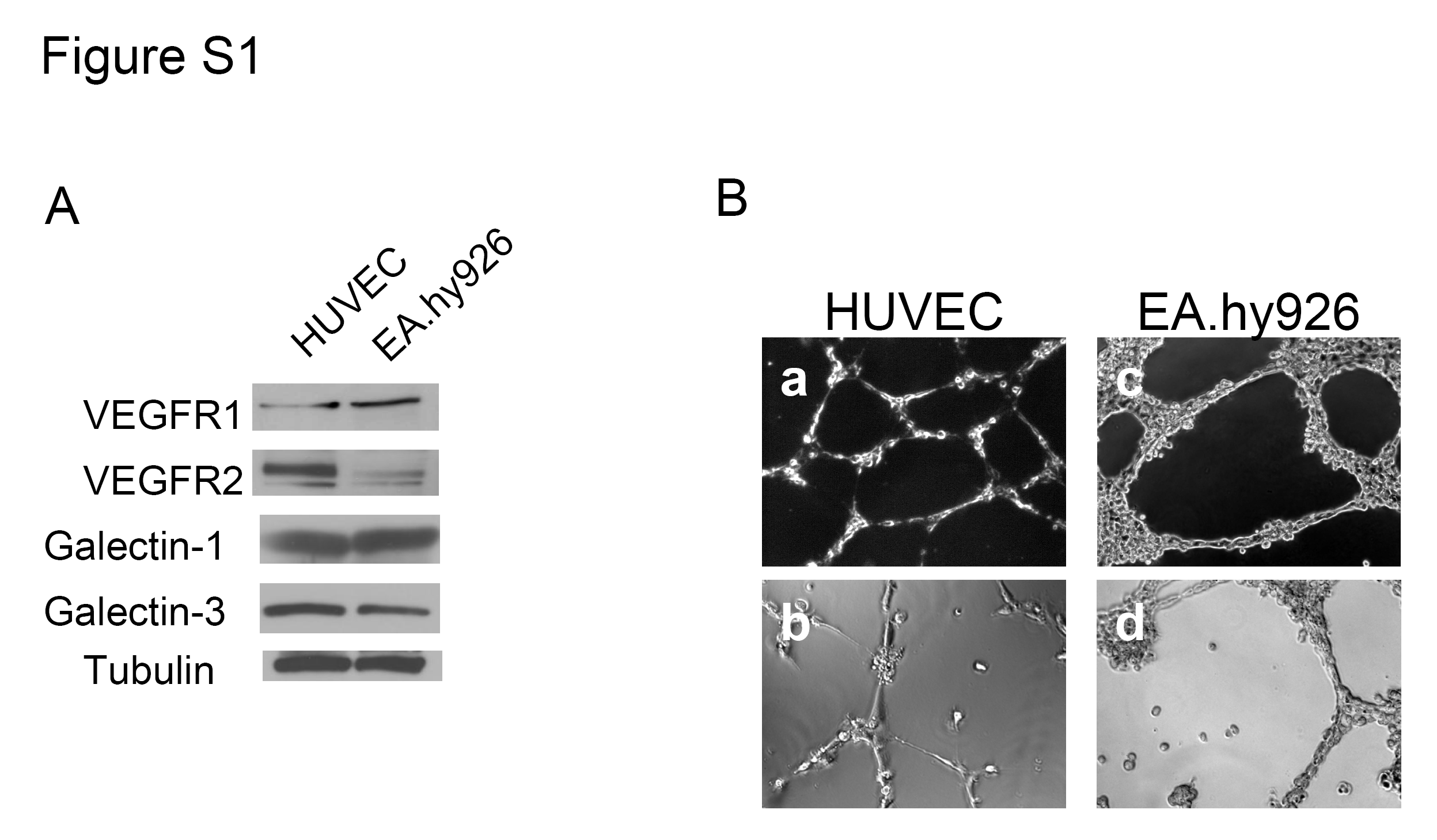

Supplement: Figure S1 — Characterisation of EA.hy926 and HUVEC cell lines. (A) Characterisation of VEGFR and galectin expression in HUVEC and EA.hy926 lysates by western blotting. Protein expression was examined using specific anti-human Abs against galectin-1 (1∶1000; PeproTech), galectin-3 (1∶1000; Novocastra, Newcastle, UK), VEGFR1 (1∶1000; Abcam) and VEGFR2 (1∶1000; Cell Signaling, Beverly, MA). Monoclonal anti-tubulin Ab (1∶5000; Abcam) served as a loading control. (B) When plated on matrigel, HUVECs and EA.hy926 cells formed capillary-like networks with different tube morphology. HUVEC tubes were thin and lined with a single cell layer, but EA.hy926 tubes were more complex, with larger diameters that were formed by clumps of cells. HUVEC tubes were characterised by dichotomous branching, but EA.hy926 tubes displayed heterogeneous branching with uneven diameters. The formation of capillary-like networks was slower for EA.hy926 cells (22 h) compared with HUVECs (6 h). (TIF) [file pone.0067029.s001.tif]

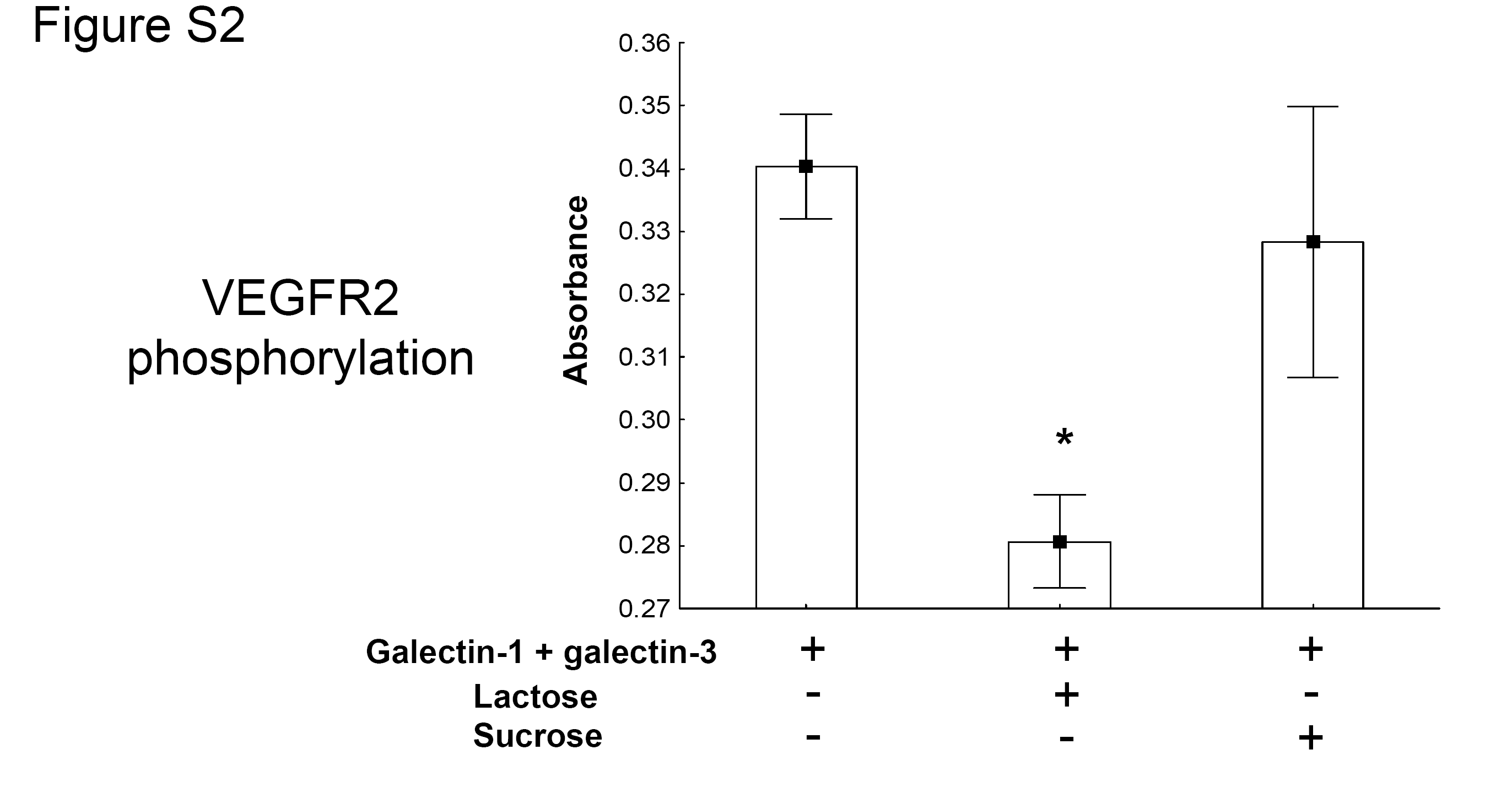

Supplement: Figure S2 — The VEGFR2 activation induced by galectin-1 and galectin-3 was inhibited by lactose but not sucrose, indicating that the effect is due to glycan binding by galectins. VEGFR2 phosphorylation levels in EA.hy926 cells following a 5-min stimulation with both galectins (1 µg/ml) in the absence or presence of lactose or sucrose (50 mmol/l). The data are presented as the mean +/− SEM (* p<0.05). (TIF) [file pone.0067029.s002.tif]
