# Supplementary material for: VEGFR1 and VEGFR2 Involvement in Extracellular Galectin-1- and Galectin-3-Induced Angiogenesis
Source: PLoS One. 2013 Jun 17;8(6):e67029. doi: 10.1371/journal.pone.0067029 (PMC3684579; doi:10.1371/journal.pone.0067029)
Supplement: Materials and Methods S1 — (DOC) [file pone.0067029.s003.doc]

**MATERIALS AND METHODS S1**

***Enzyme-Linked Immunosorbent Assays***

The levels of phosphorylated VEGFR1, VEGFR2, extracellular signal-regulated kinase (ERK)1/2, heat-shock protein 27 (Hsp27), Src, protein kinase B (Akt) and focal adhesion kinase (FAK) were examined using human phospho-VEGFR1, phospho-VEGFR2, phospho-ERK1/2, phospho-Hsp27, phospho-Src, phospho-Akt and phospho-FAK DuoSet IC ELISAs (R&D Systems). EA.hy926 cells were treated with or without galectin-1, galectin-3, or both at 1 µg/ml (each) for 5 min (for VEGFR1 and VEGFR2 assays) or 10 min (for the other assays). Subsequently, the cells were lysed according to the manufacturer’s protocol. The total protein concentration was determined using Bio-Rad protein assay dye reagents, and the results were normalised by protein concentration. An immobilised capture antibody specific to each target bound to the total respective protein, and unbound material was removed by washing. Phosphorylated proteins were detected using an HRP-conjugated detection antibody that was specific for phosphorylated tyrosines (VEGFR1 and VEGFR2) or specifically targeted a phosphorylated protein (for the other proteins). Incubation with a developing solution provided a quantitative colorimetric readout.

***Proximity ligation assay***

We used the Duolink in situ PLA kit from Olink Bioscience (Olink Bioscience, Uppsala, Sweden) to detect colocalisation between VEGFR1 or VEGFR2 and early endosome antigen-1 (EEA1). Briefly, EA.hy926 cells and HUVECs (50 000 cells/ml) were plated on LabTek slides (Nunc, VWR, Leuven, Belgium). After 24 h, the cells were left untreated or treated with galectin-1, -3 or -1 and -3 at 1 µg/ml (each) for 2 h. After fixation in paraformaldehyde, the cells were incubated with the following primary antibodies: mouse mAb against EEA1 (1:100; B&D Biosciences) and rabbit mAb against either VEGFR2 (1:100; Cell Signaling) or VEGFR1 (1/100, Abcam). The Duolink system provides oligonucleotide-labelled secondary antibodies (PLA probes) for each of the primary antibodies that, in combination with a DNA amplification-based reporter system, generate a signal only when the two primary antibodies are in close proximity. The signal from each detected pair of primary antibodies was visualised as a PLA signal (as detailed in the manufacturer's instructions).

The slides were evaluated using an IX81 motorised inverted research microscope (Olympus, Aartselaar, Belgium). A z-stack of 7 fluorescent micrographs was obtained with the 60 × objective for each observed field. Additionally, a single phase-contrast image was obtained of the same field. The cell images were stored in 16-bit TIF format for quantitative analysis. The determination of the PLA signal per cell was obtained with an image analysis tool developed by the Laboratory of Image Synthesis and Analysis. Briefly, the image stacks presented some (unidentified) noise signal around the nucleus of the cell. This noise had to be filtered out to detect the dots automatically. Each PLA dot was clearly apparent on a single z-slice only, whereas the noise signal was apparent on all z-slices. The filtering method projected the z-stack into a single image with a reinforced dot signal and attenuated noise signal. The theory behind this projection is that the distribution of values along the z axis has a higher variance at (x,y) positions where a dot is present (due to the intensity peak on a single z-slice) than where there is consistent noise or background across all slides. A threshold operation was then applied to the projected image to produce a binary image, which defined the detected dots. After this automatic detection, the software presented the user with the phase-contrast image of the observed field, prompting them to outline each cell. All of the detected dots inside a given outline were counted as PLA signals for that cell.
