# Supplementary figures and images for: Correction: VEGFR1 and VEGFR2 Involvement in Extracellular Galectin-1- and Galectin-3-Induced Angiogenesis
Source: PLoS One. 2023 Dec 7;18(12):e0295736. doi: 10.1371/journal.pone.0295736 (PMC10703273; doi:10.1371/journal.pone.0295736)

## Slide 1
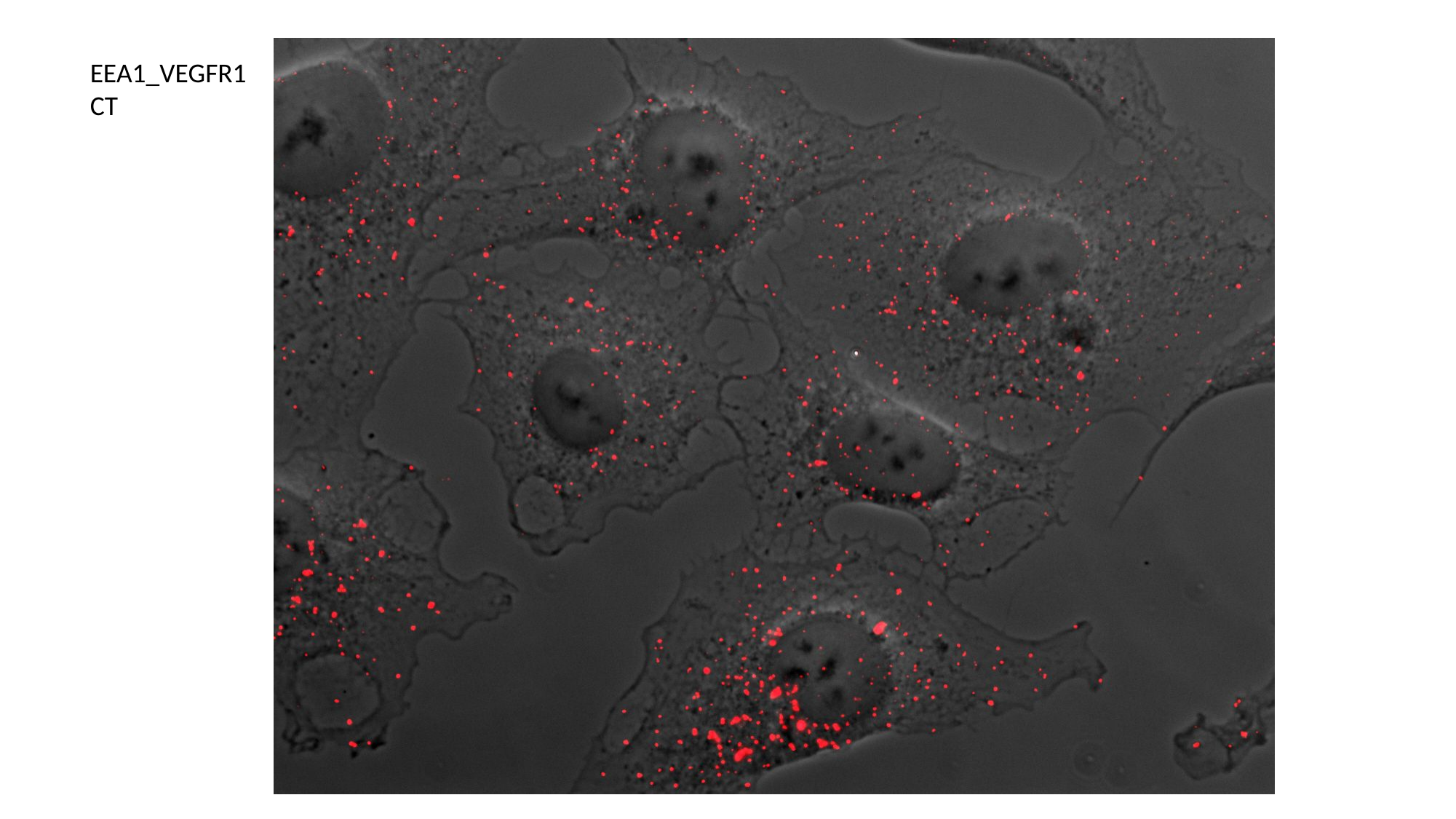

EEA1_VEGFR1
CT

## Slide 2
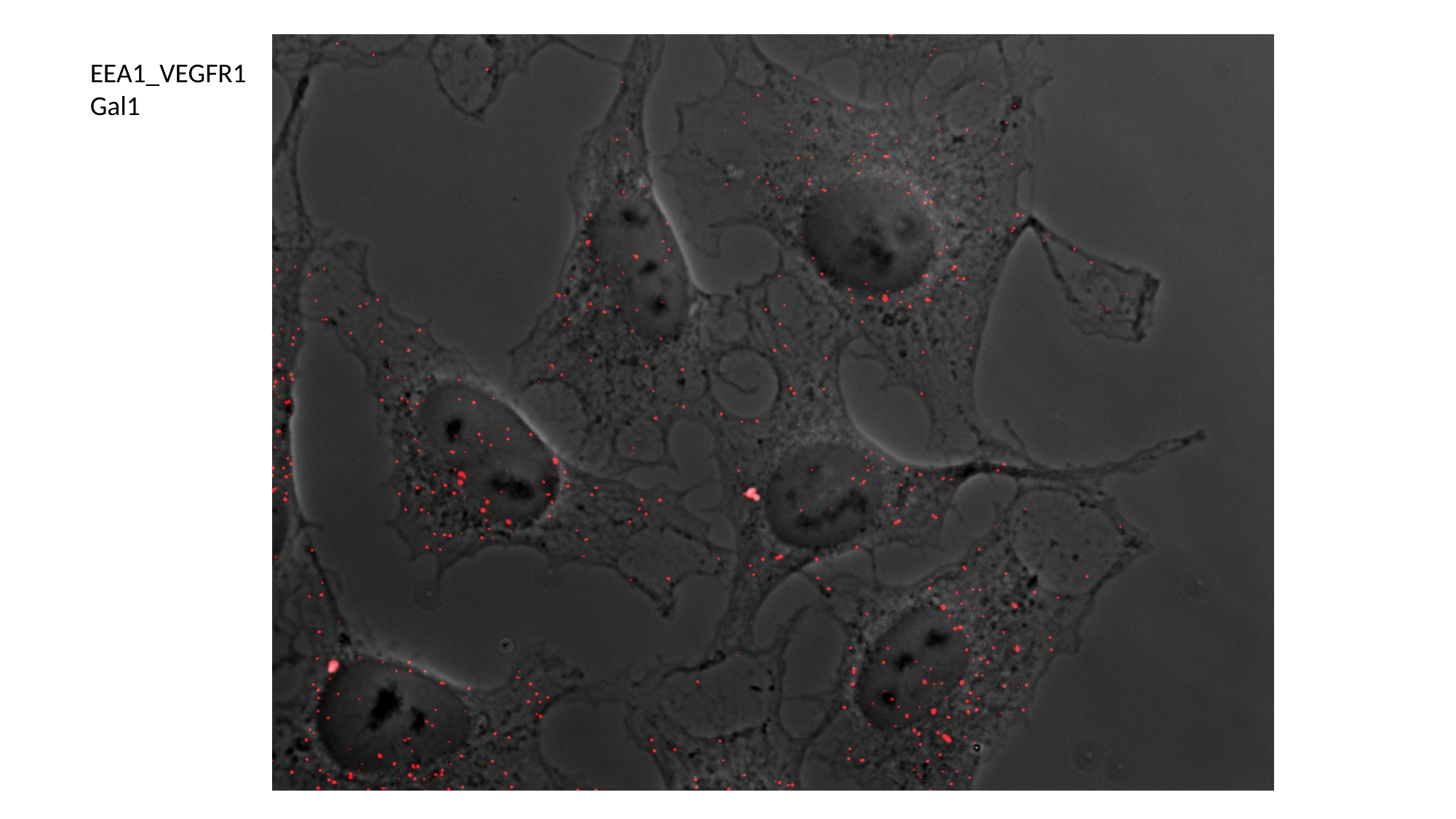

EEA1_VEGFR1
Gal1

## Slide 3
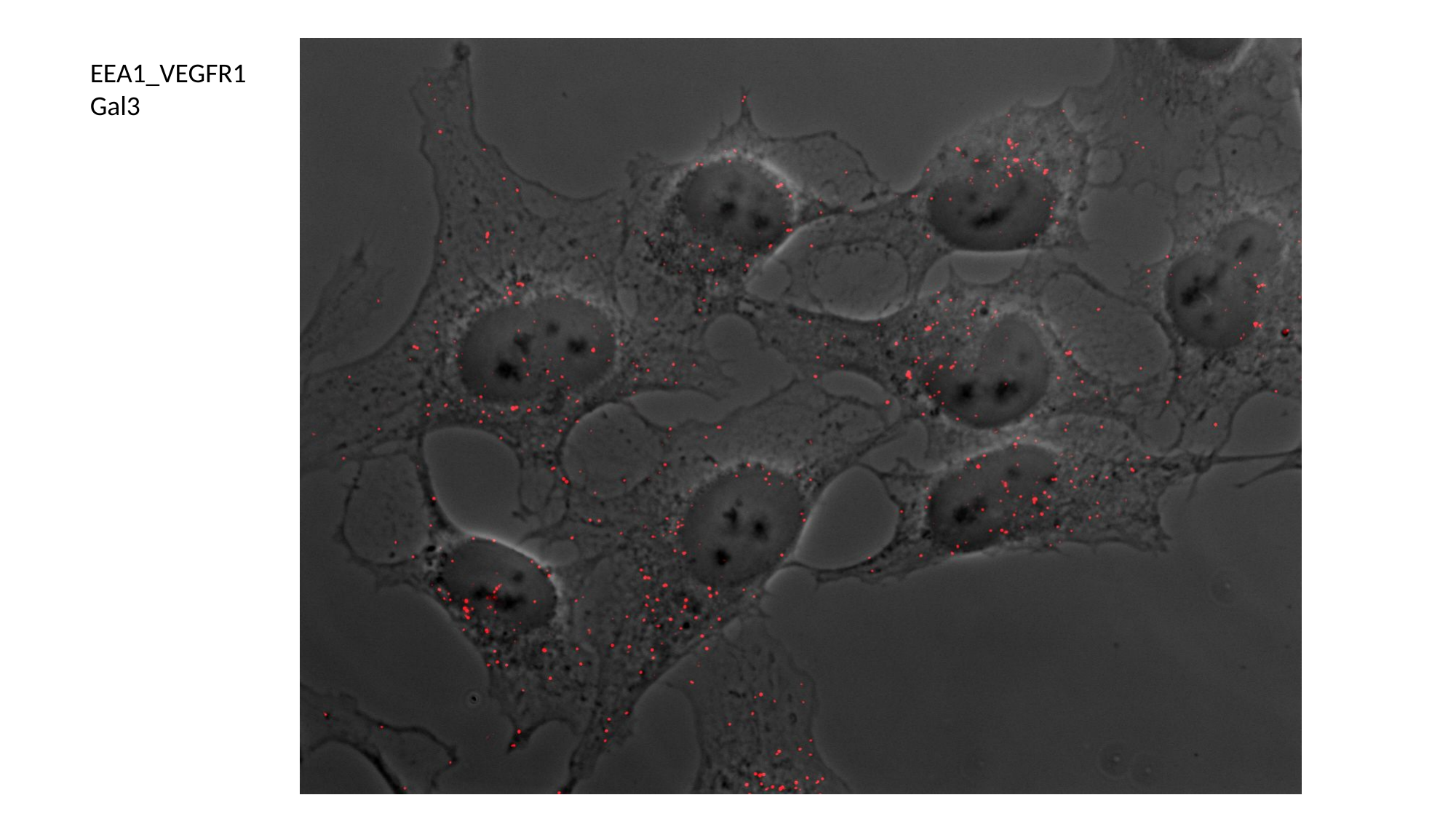

EEA1_VEGFR1
Gal3

## Slide 4
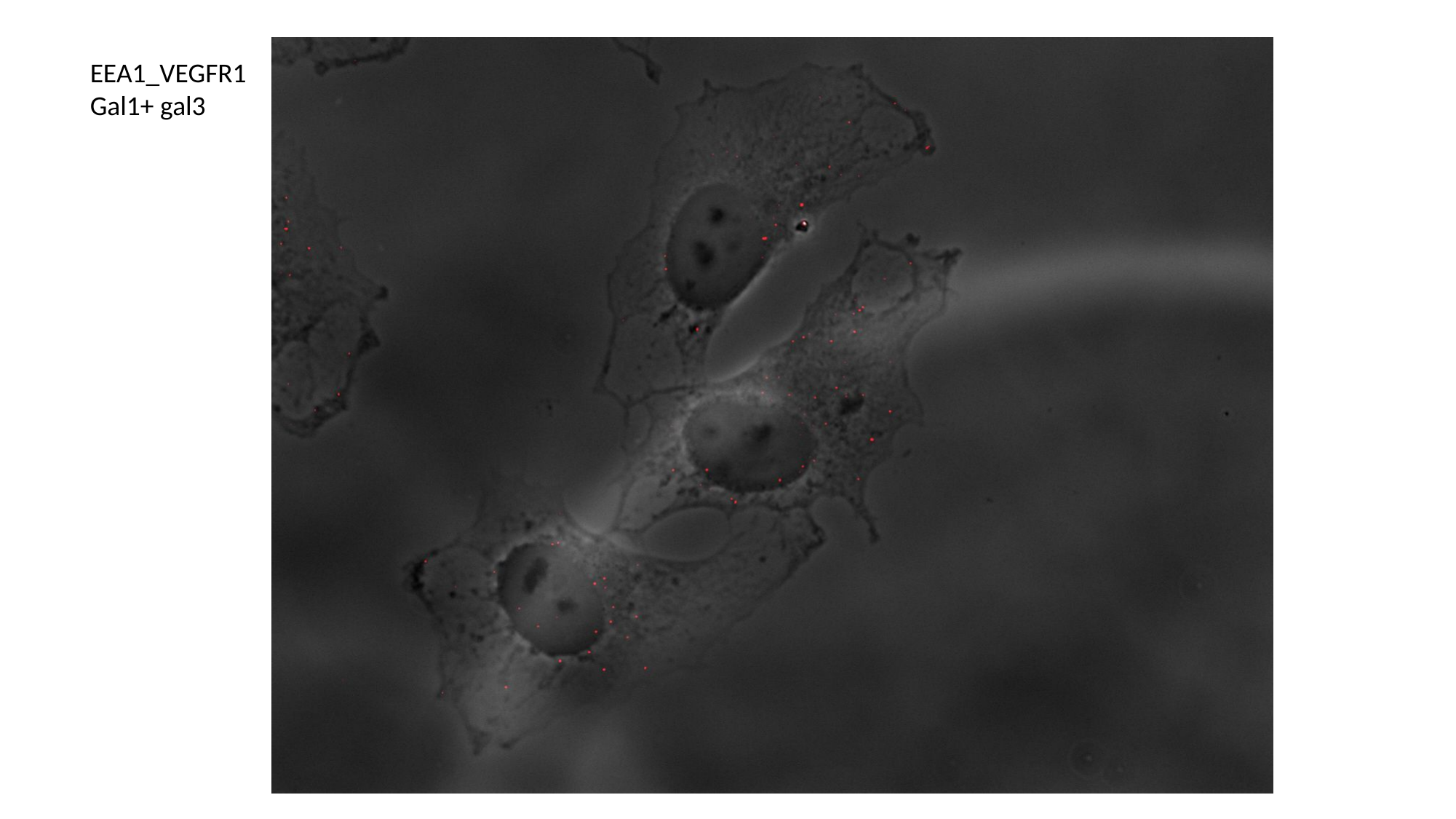

EEA1_VEGFR1
Gal1+ gal3

Supplement: S1 File — (ZIP) [file pone.0295736.s001.zip › S1 File/FIG5A.pptx]

## Slide 1
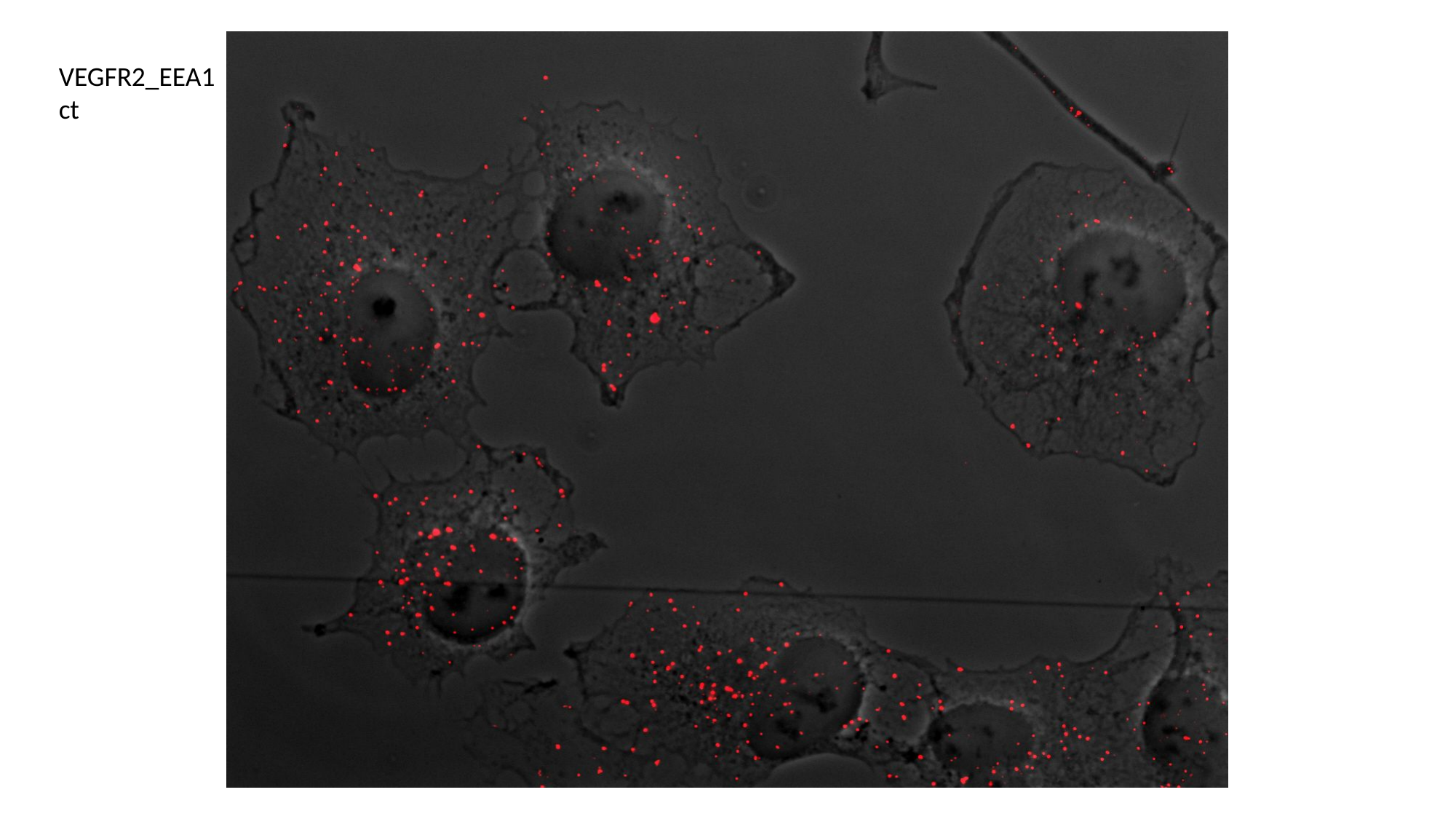

VEGFR2_EEA1
ct

## Slide 2
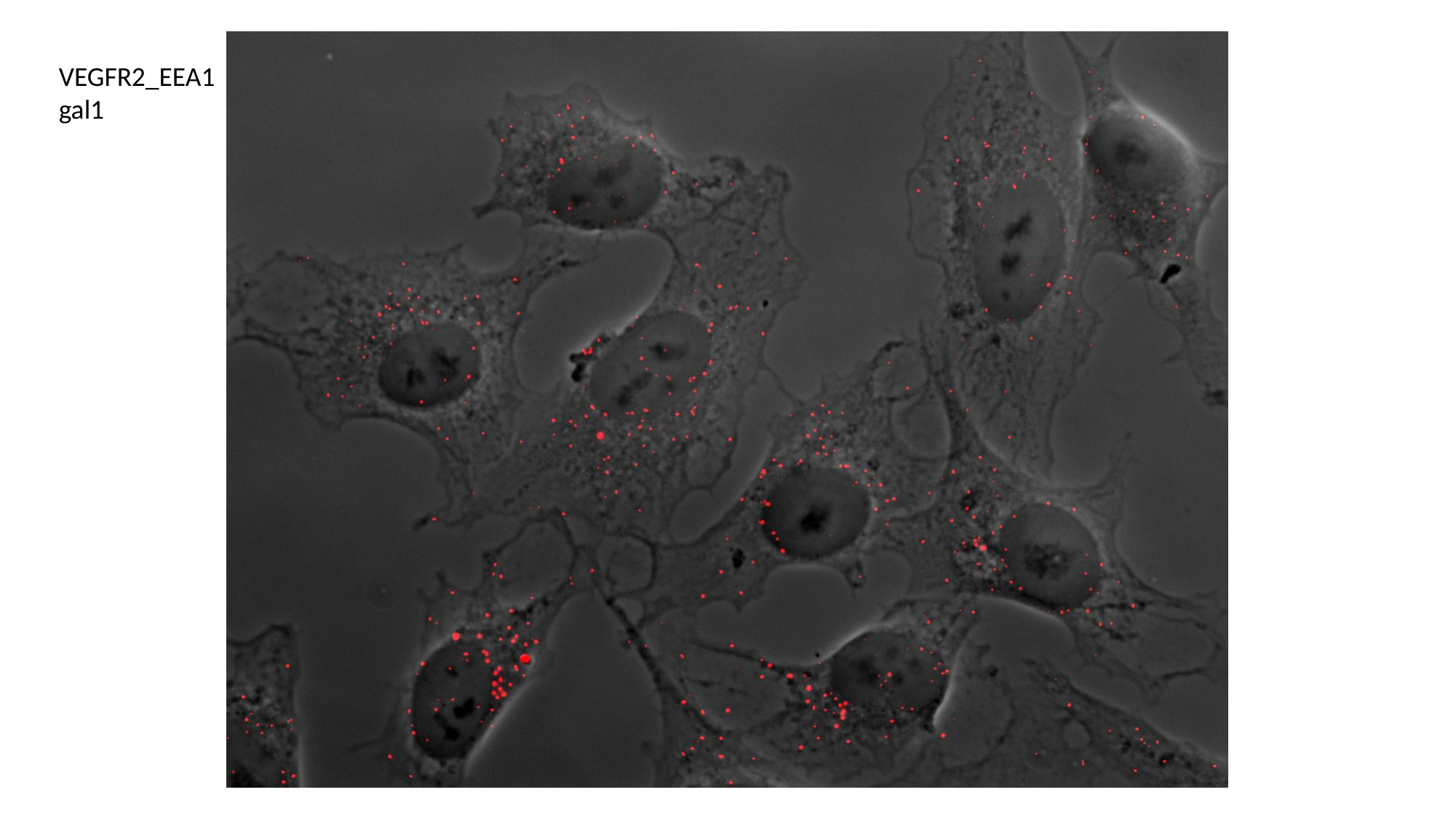

VEGFR2_EEA1
gal1

## Slide 3
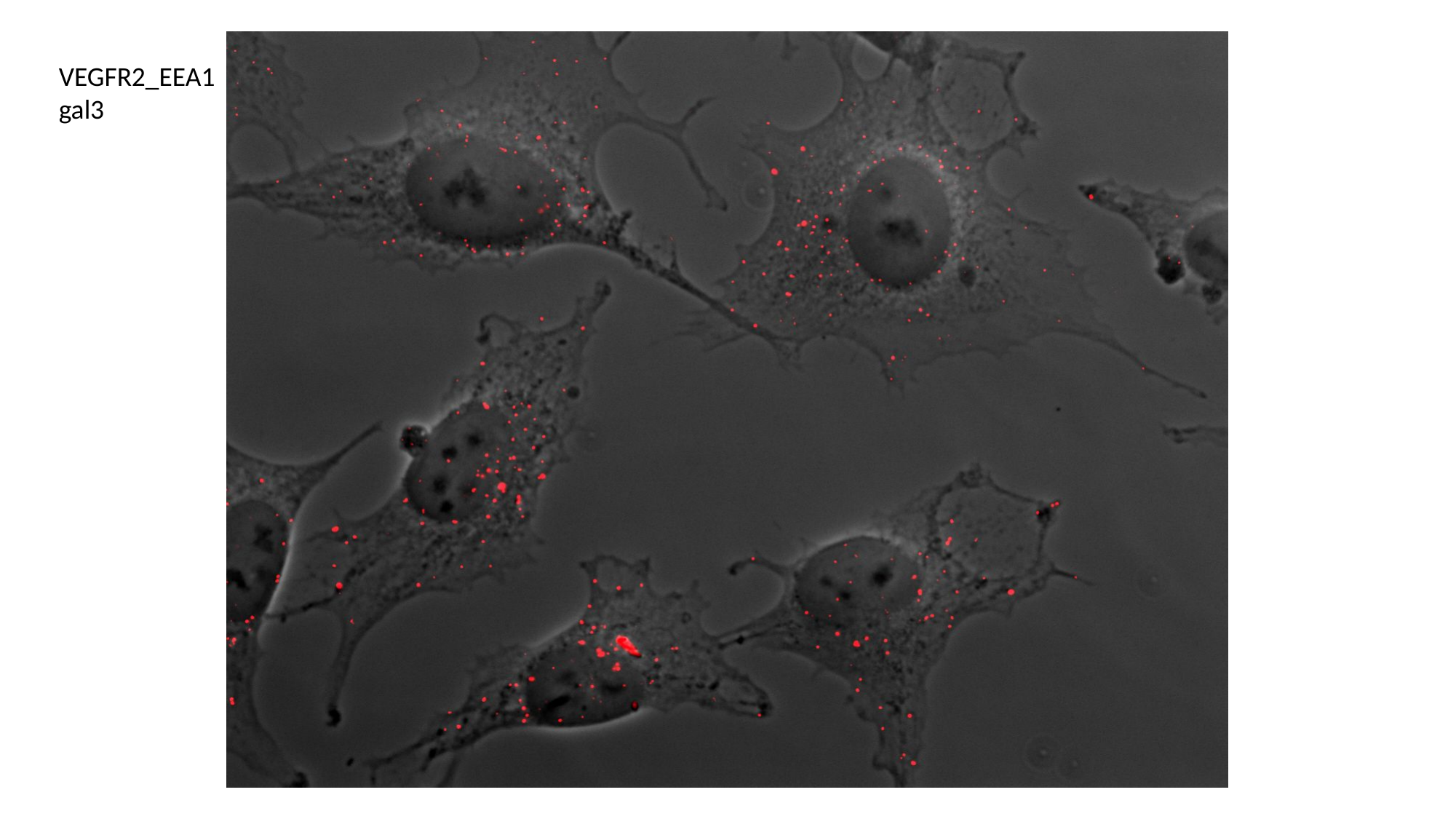

VEGFR2_EEA1
gal3

## Slide 4
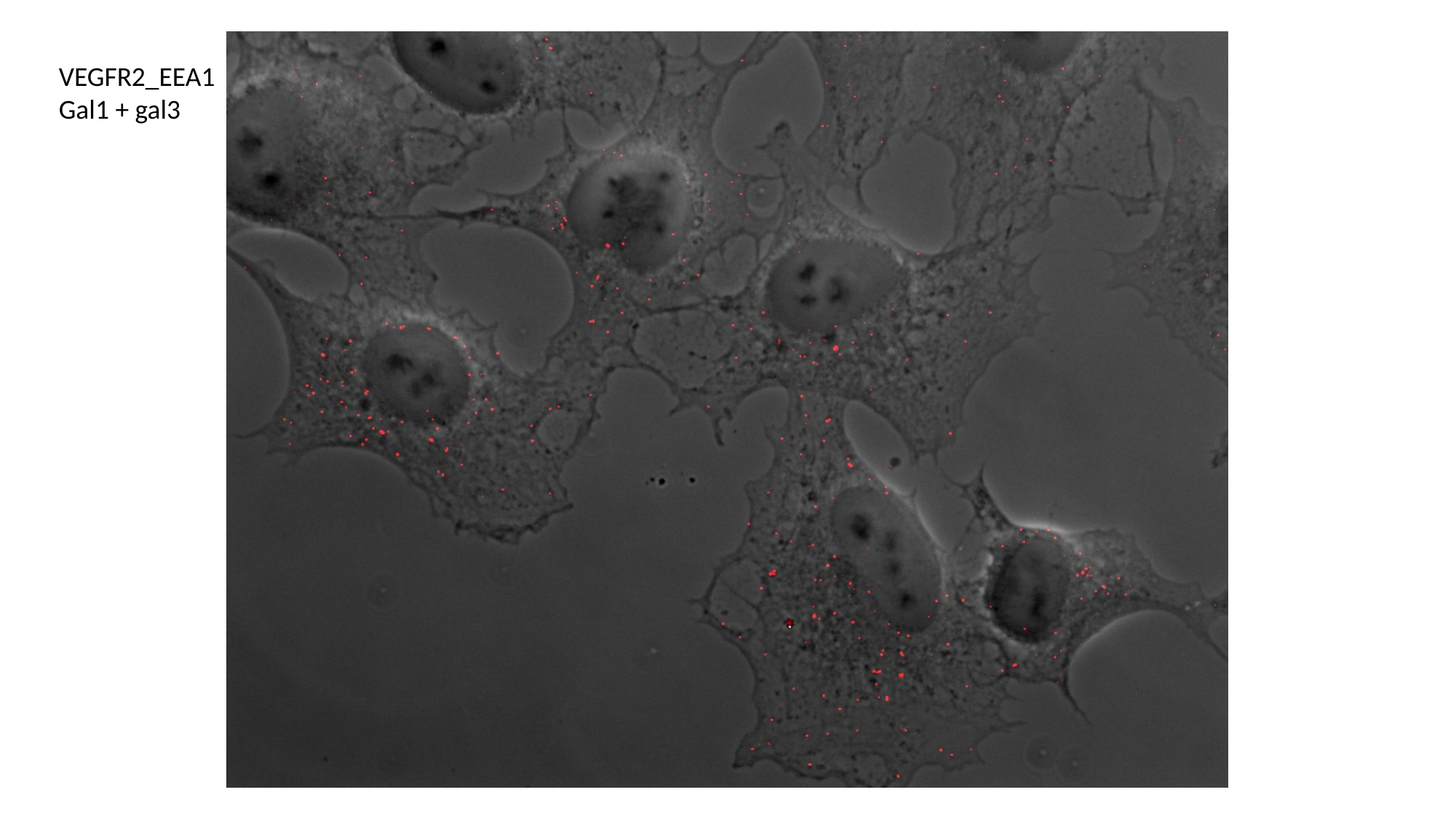

VEGFR2_EEA1
Gal1 + gal3

Supplement: S1 File — (ZIP) [file pone.0295736.s001.zip › S1 File/FIG5B.pptx]

## Slide 1
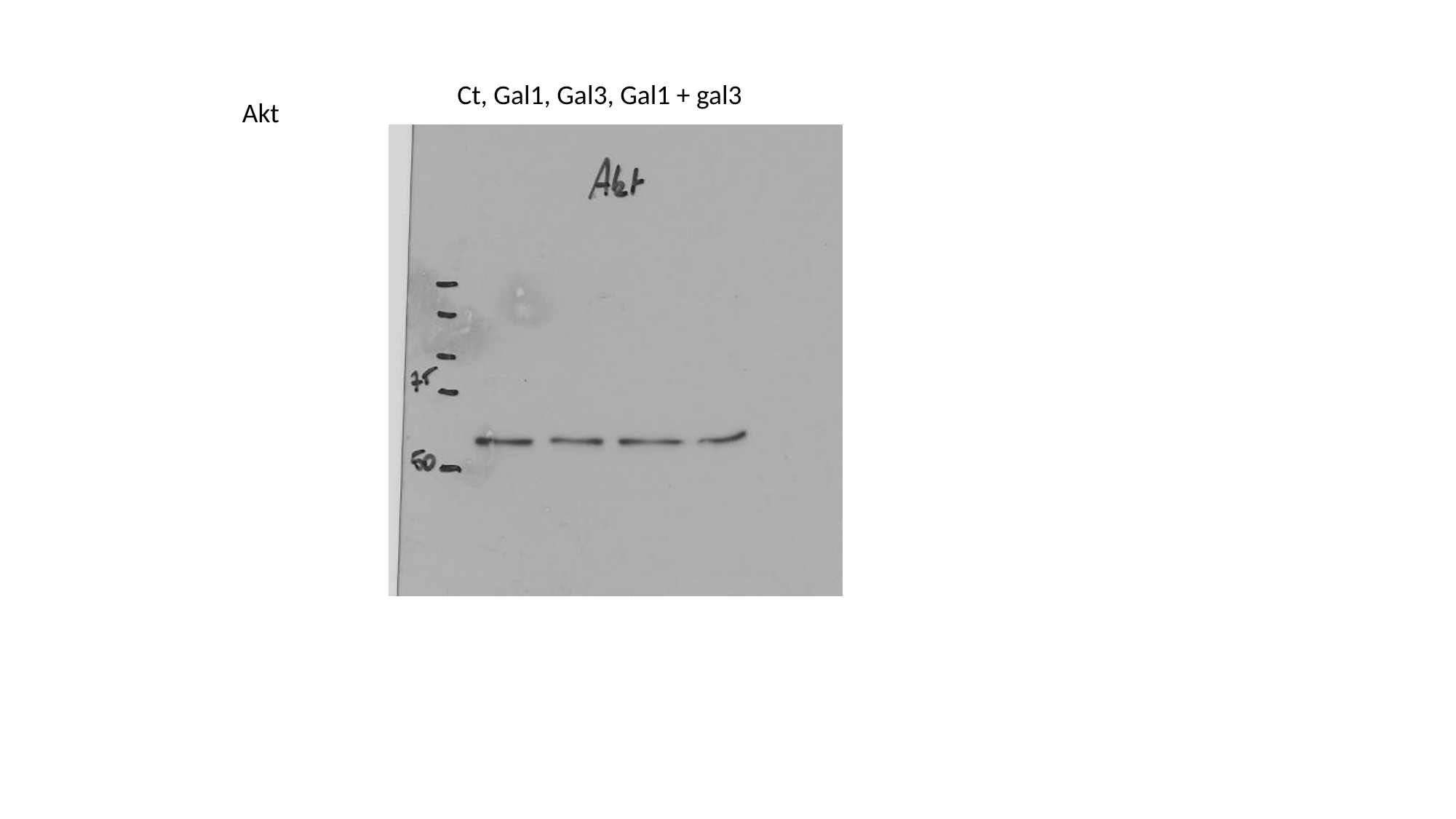

Ct, Gal1, Gal3, Gal1 + gal3
Akt

## Slide 2
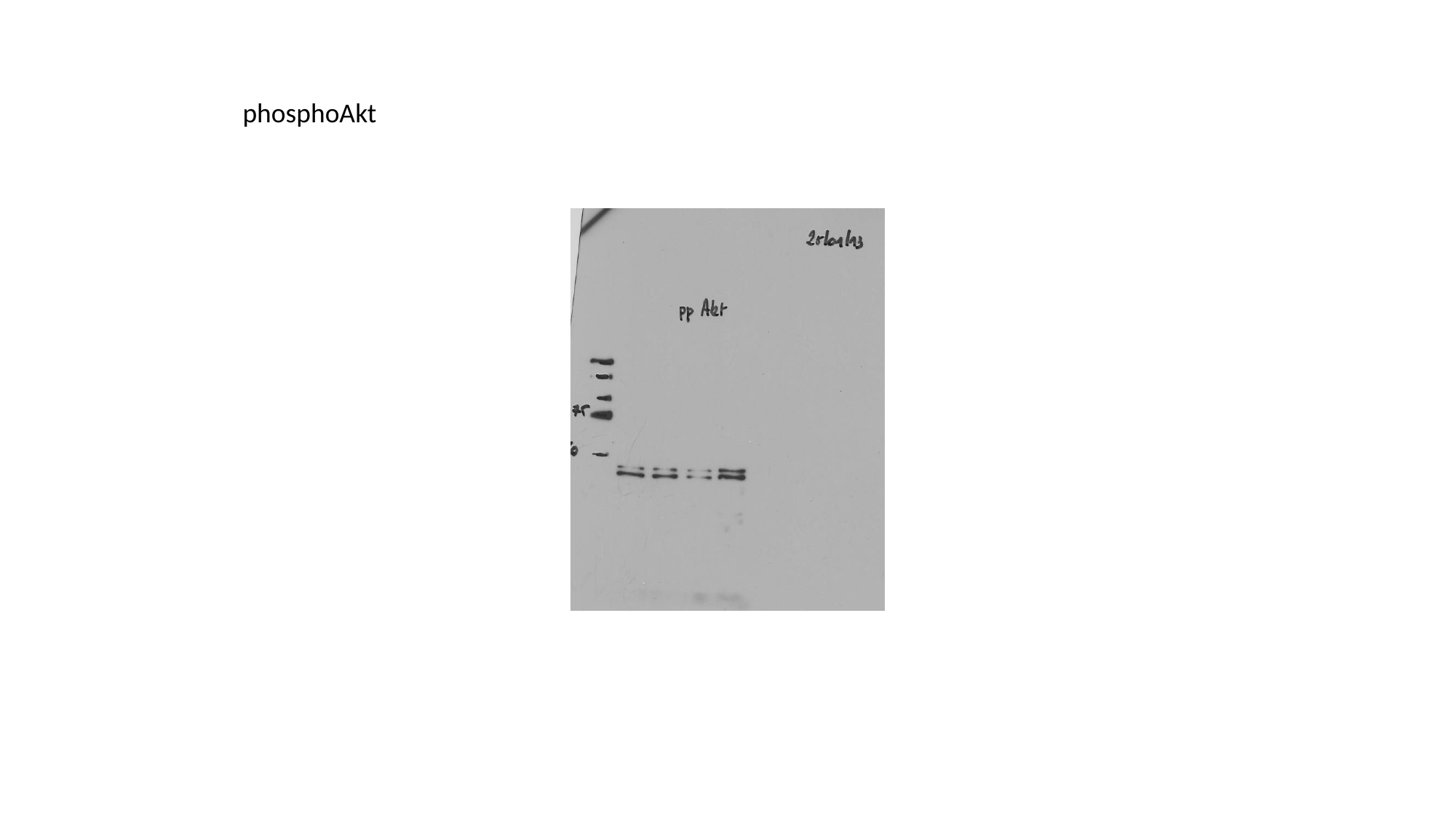

phosphoAkt

## Slide 3
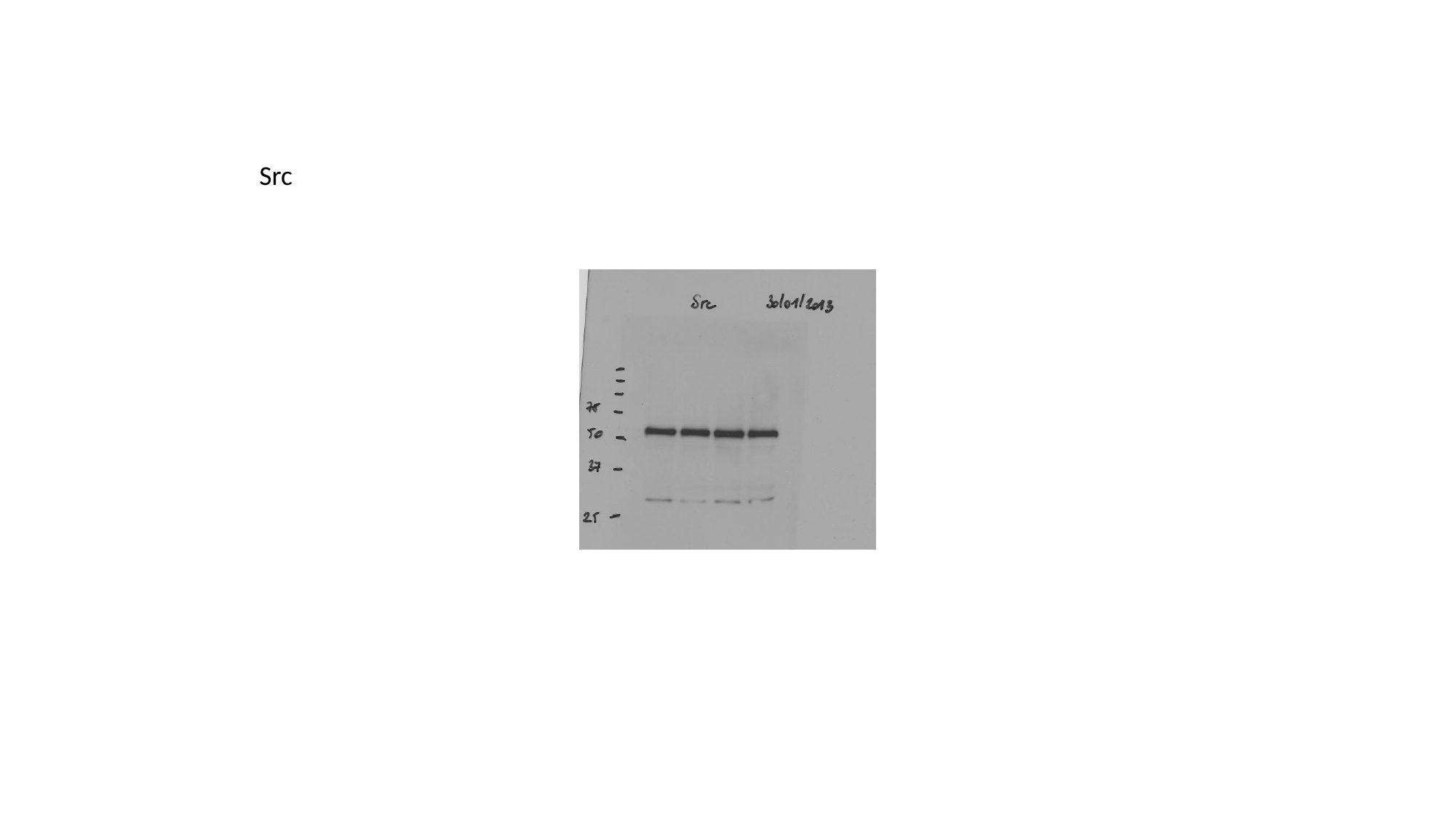

Src

## Slide 4
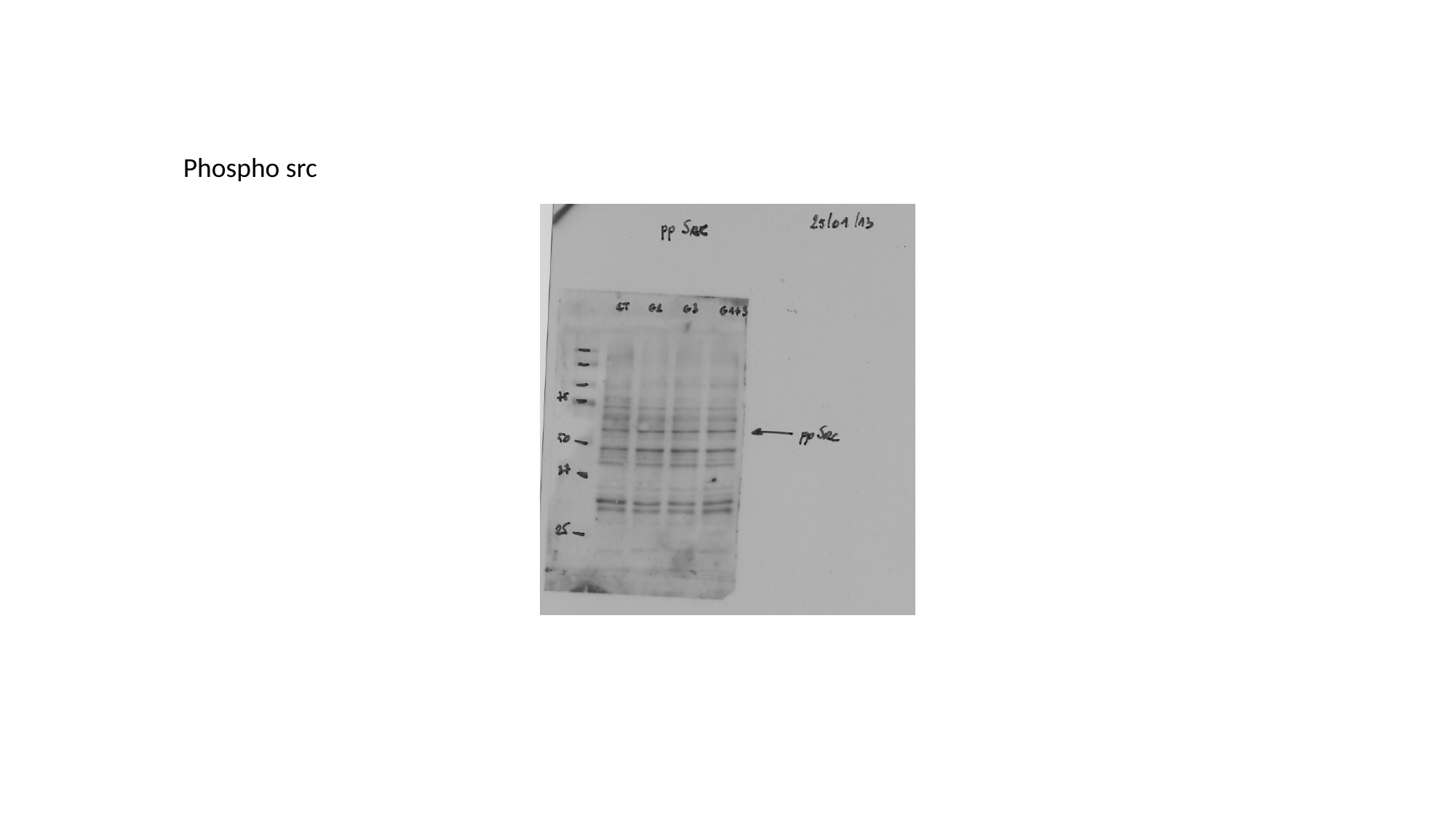

Phospho src

Supplement: S4 File — The addition of galectin-1, galectin-3 or both galectins together had no effect on Akt or Src protein expression or Src phosphorylation evaluated by Western blot. (PPTX) [file pone.0295736.s004.pptx]
