# Supplementary material for: Correction: VEGFR1 and VEGFR2 Involvement in Extracellular Galectin-1- and Galectin-3-Induced Angiogenesis
Source: PLoS One. 2023 Dec 7;18(12):e0295736. doi: 10.1371/journal.pone.0295736 (PMC10703273; doi:10.1371/journal.pone.0295736)
Supplement: S3 File — Tube formation was maximal after 22 h at the concentration of 12×103 cells/well for EA.hy926 cells. (ZIP) [file pone.0295736.s003.zip › S3 File/Matrigelsettingup_illustration.pptx]

## Slide 1
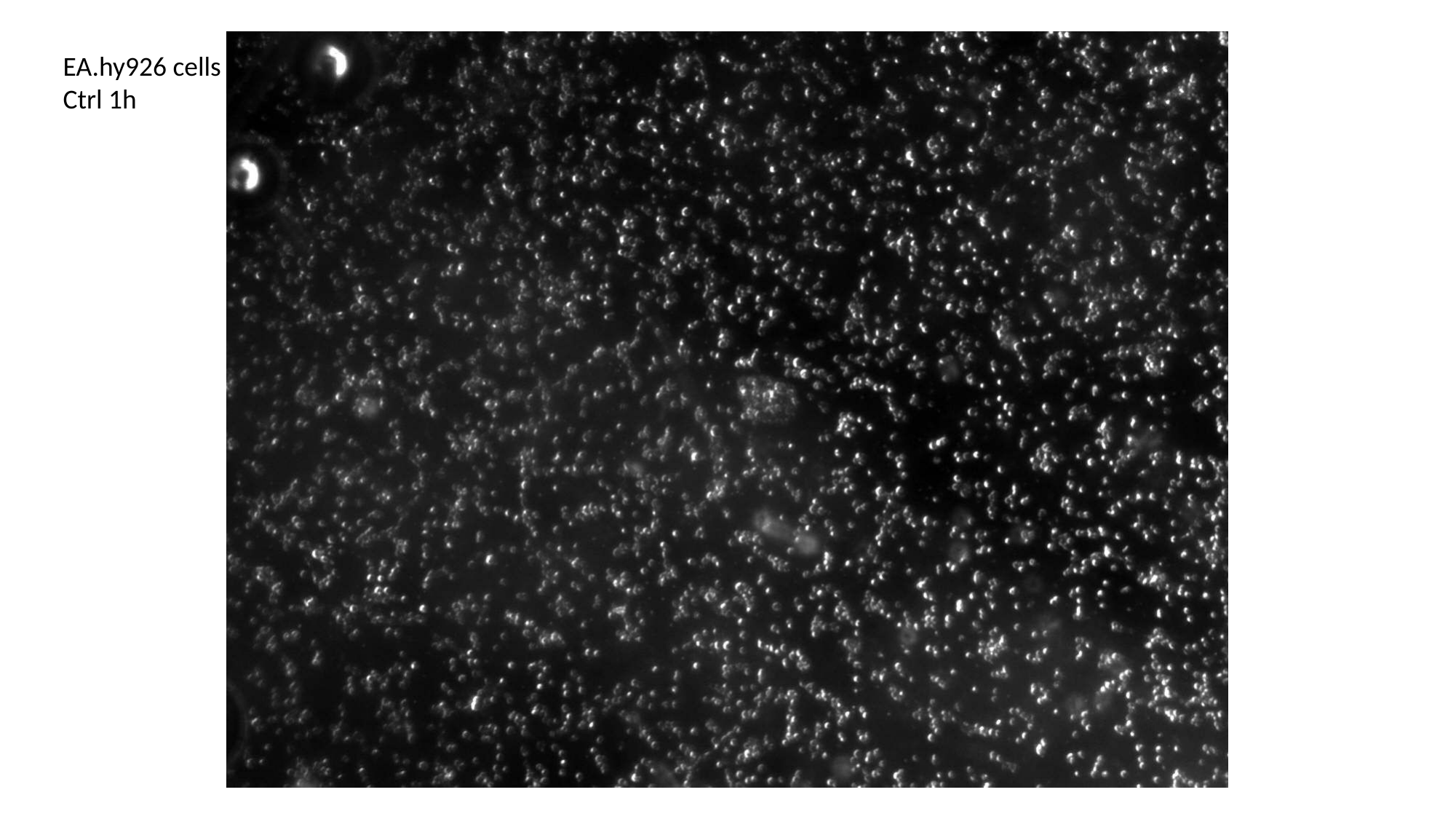

EA.hy926 cells
Ctrl 1h

## Slide 2
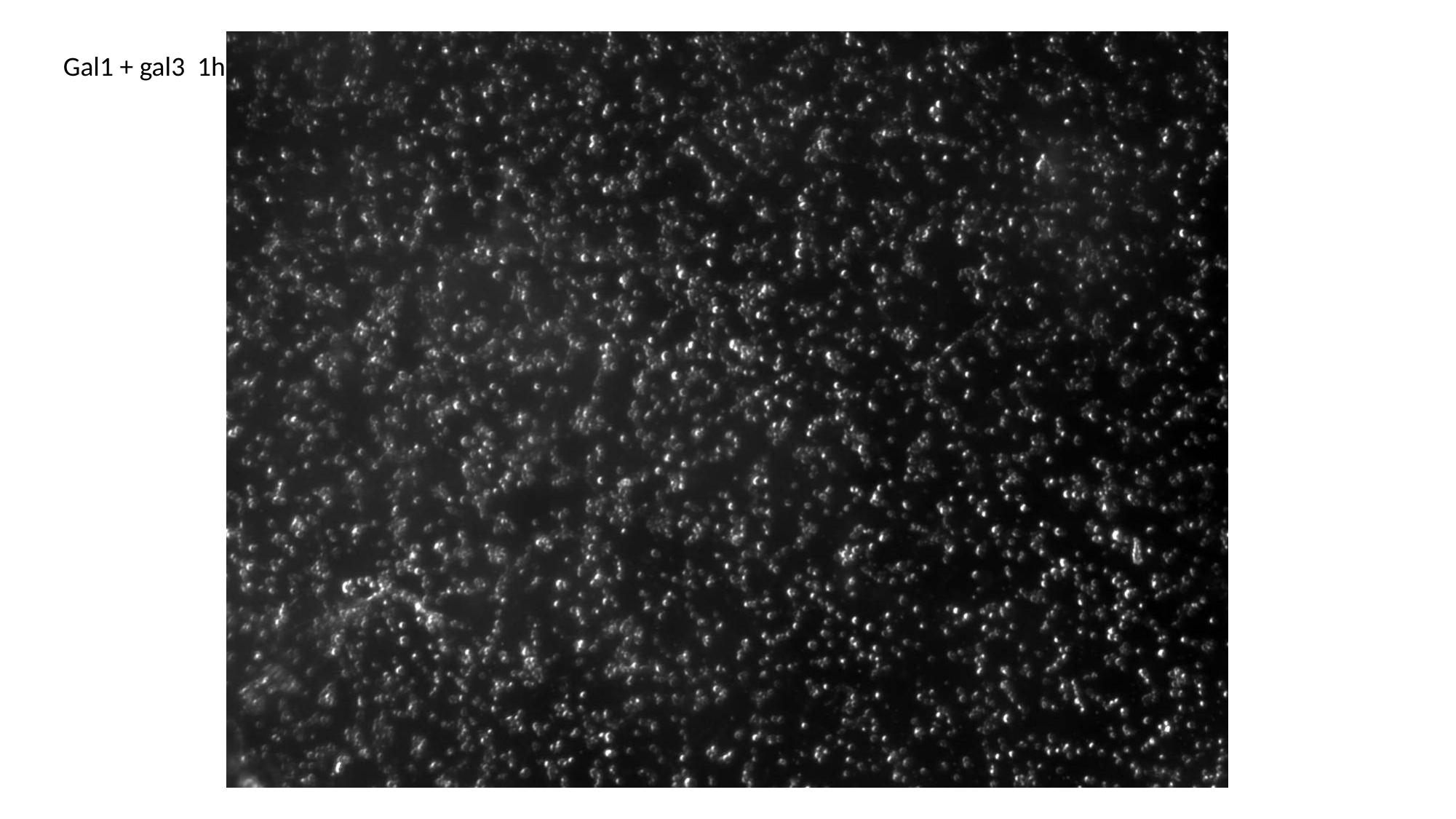

Gal1 + gal3 1h

## Slide 3
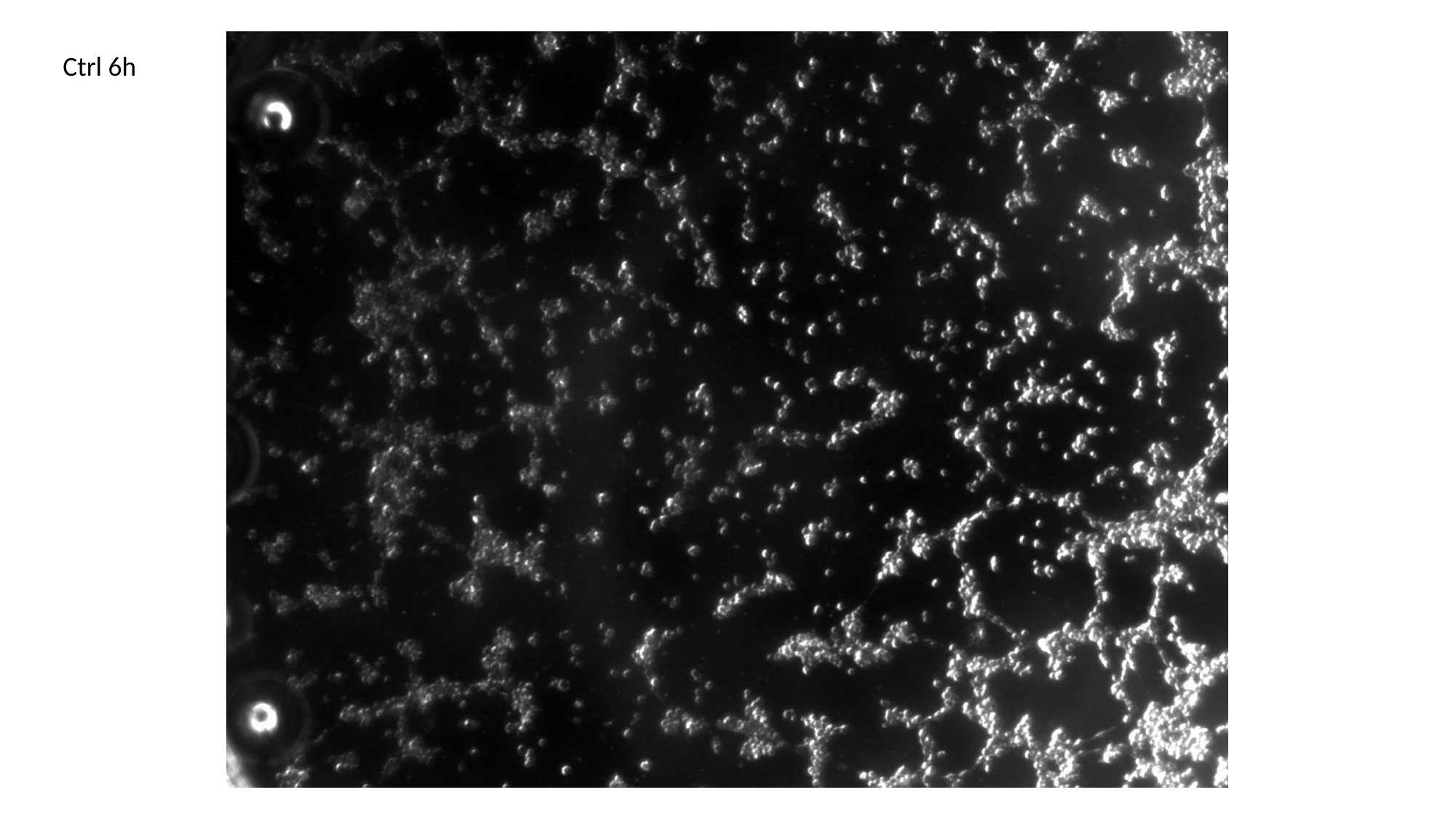

Ctrl 6h

## Slide 4
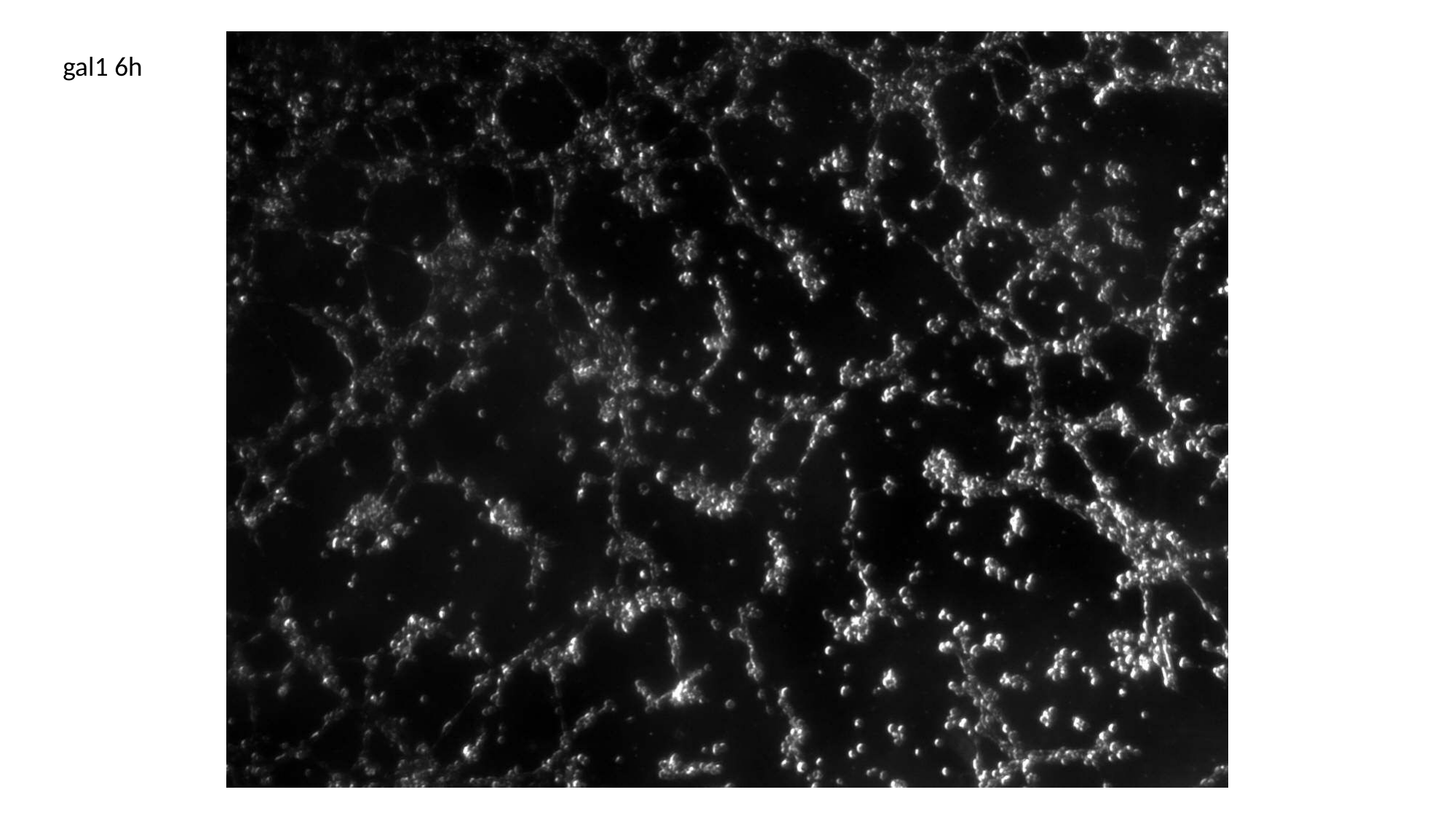

gal1 6h

## Slide 5
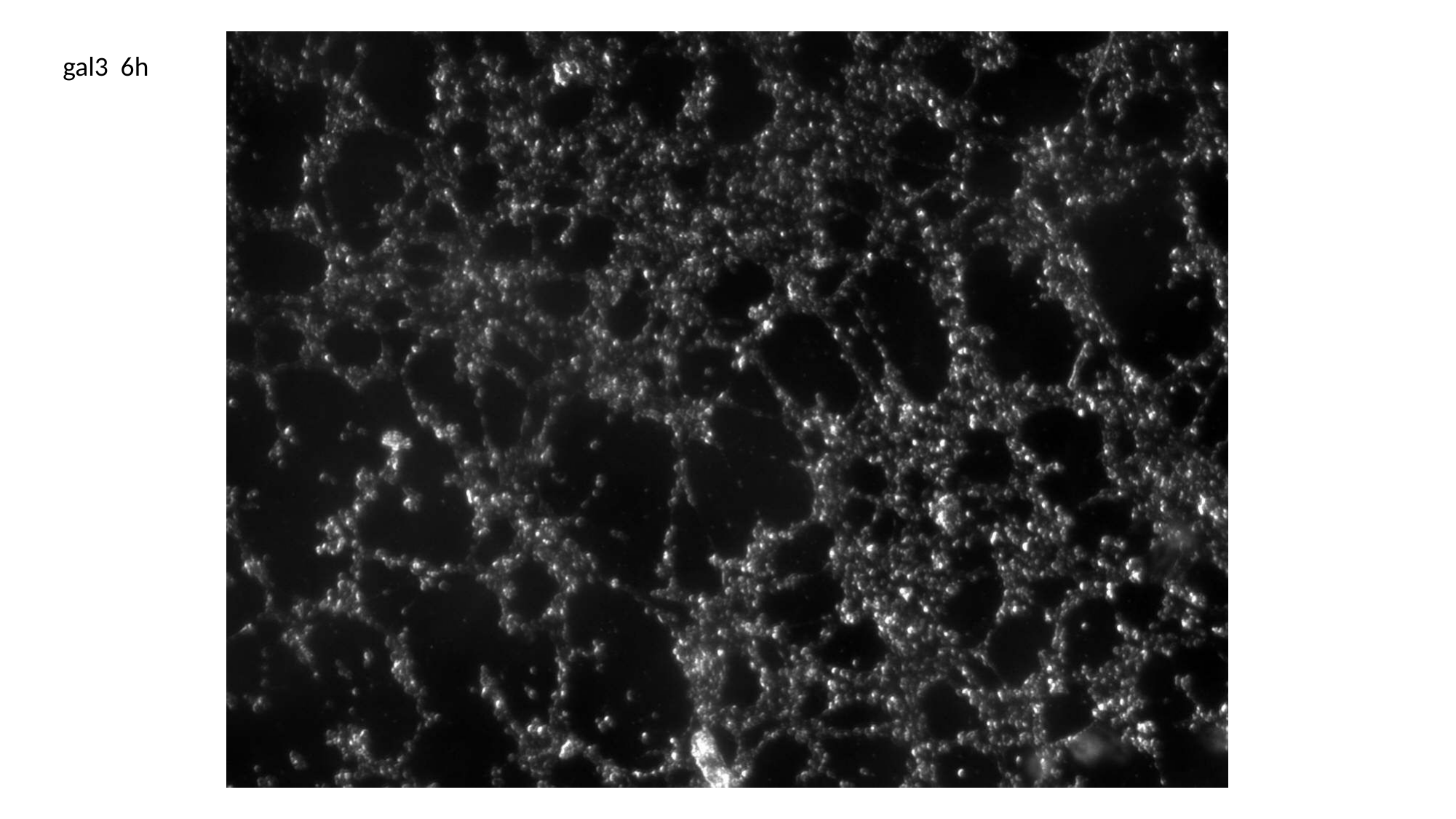

gal3 6h

## Slide 6
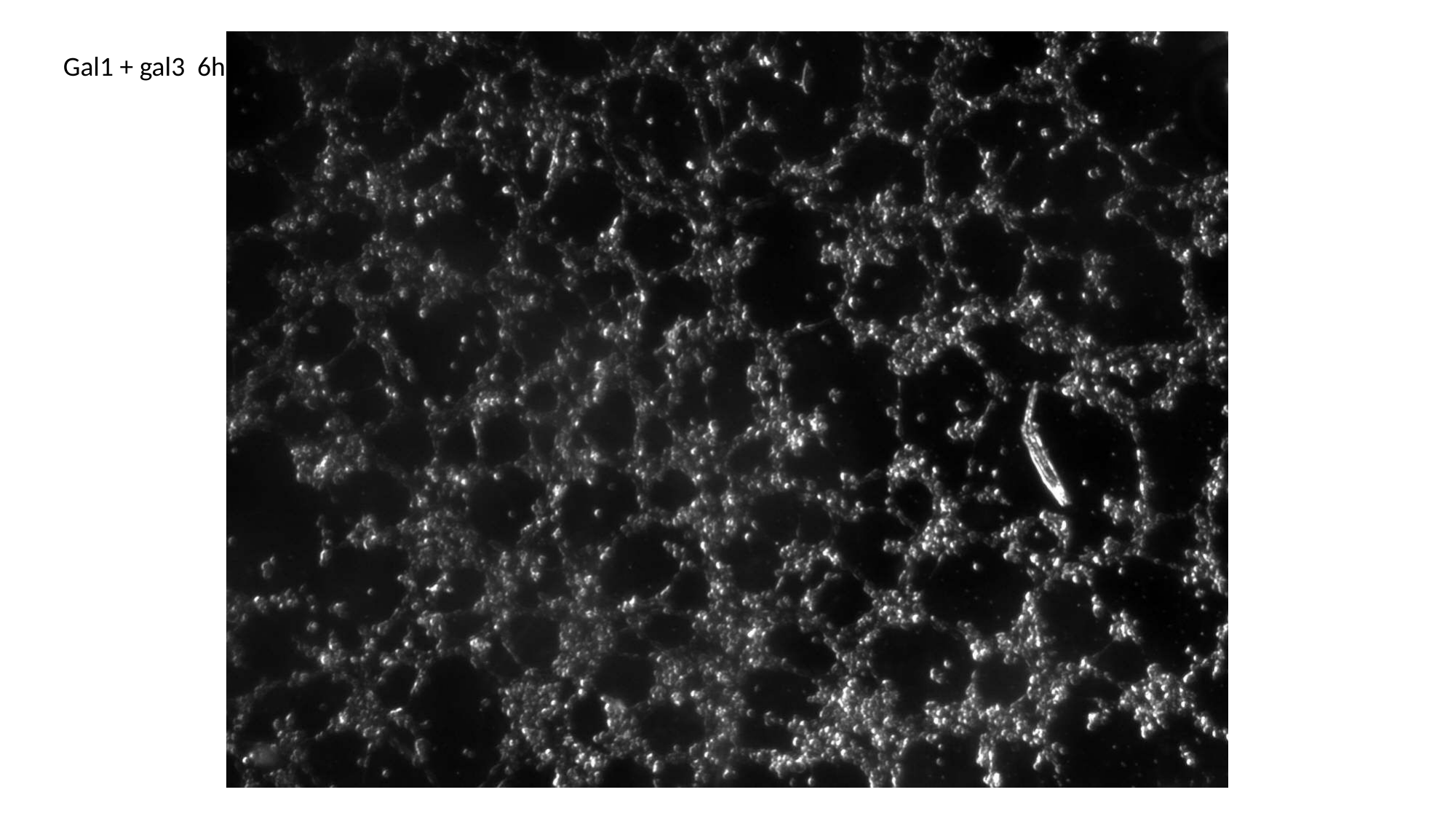

Gal1 + gal3 6h

## Slide 7
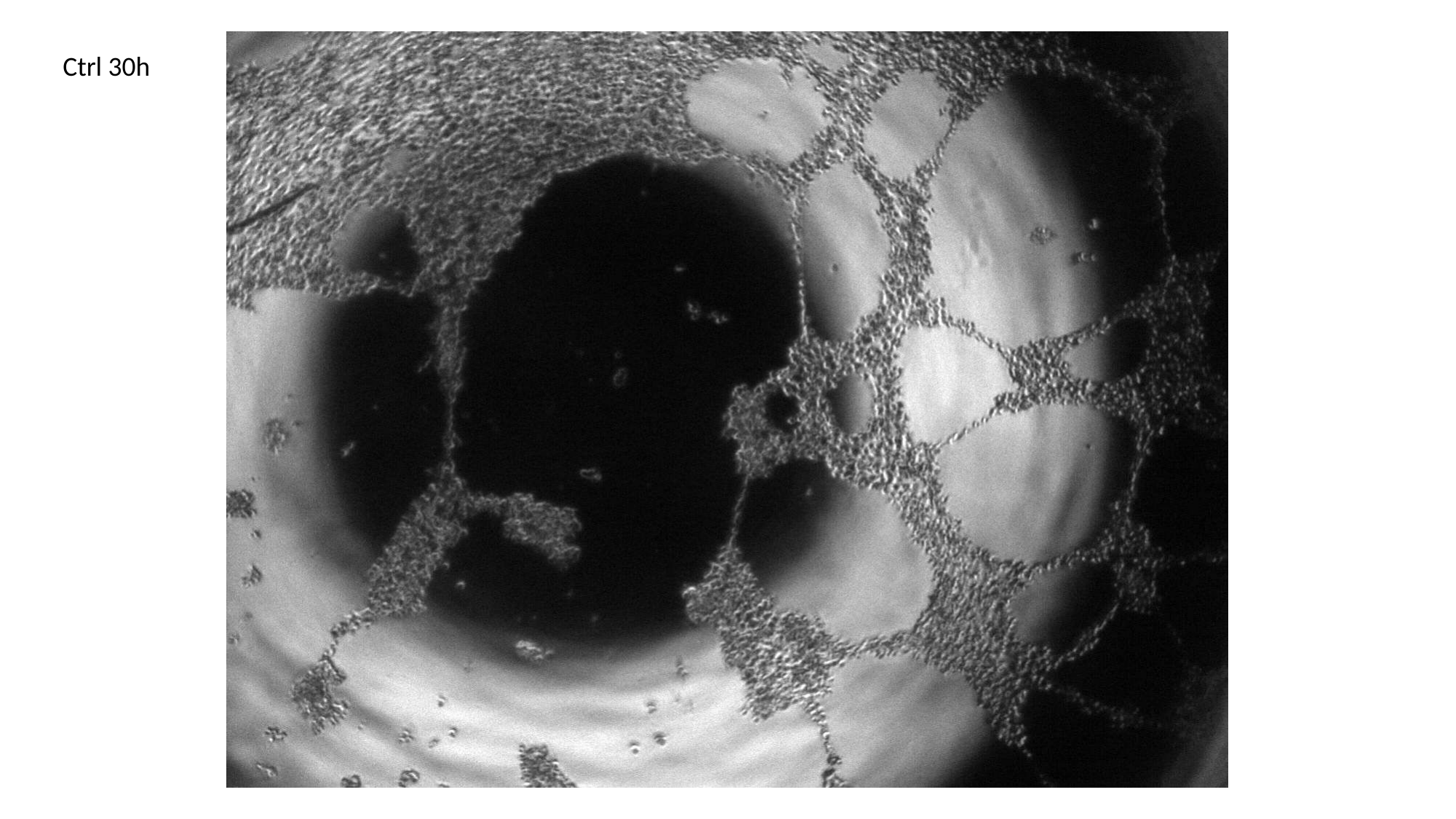

Ctrl 30h

## Slide 8
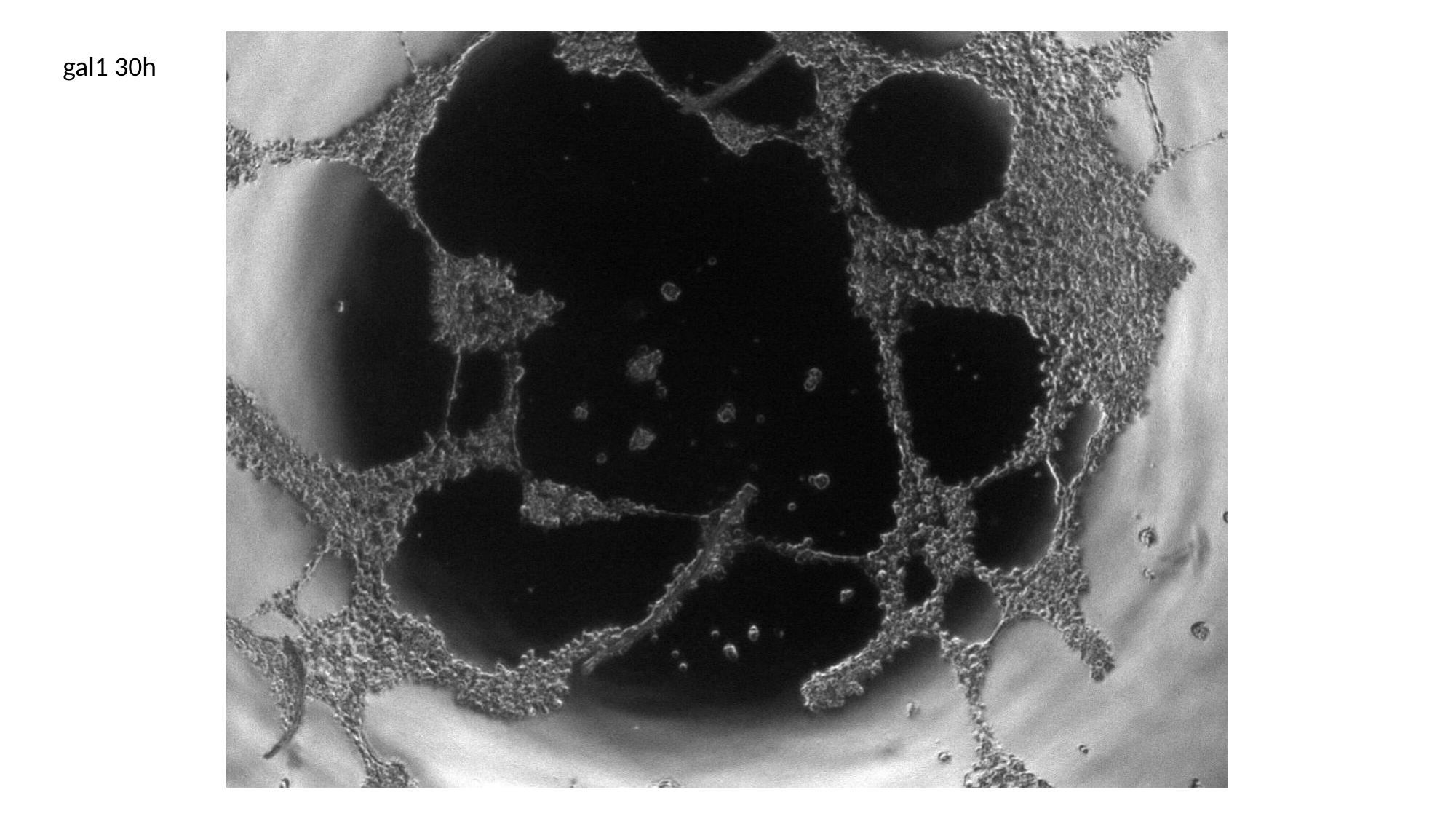

gal1 30h

## Slide 9
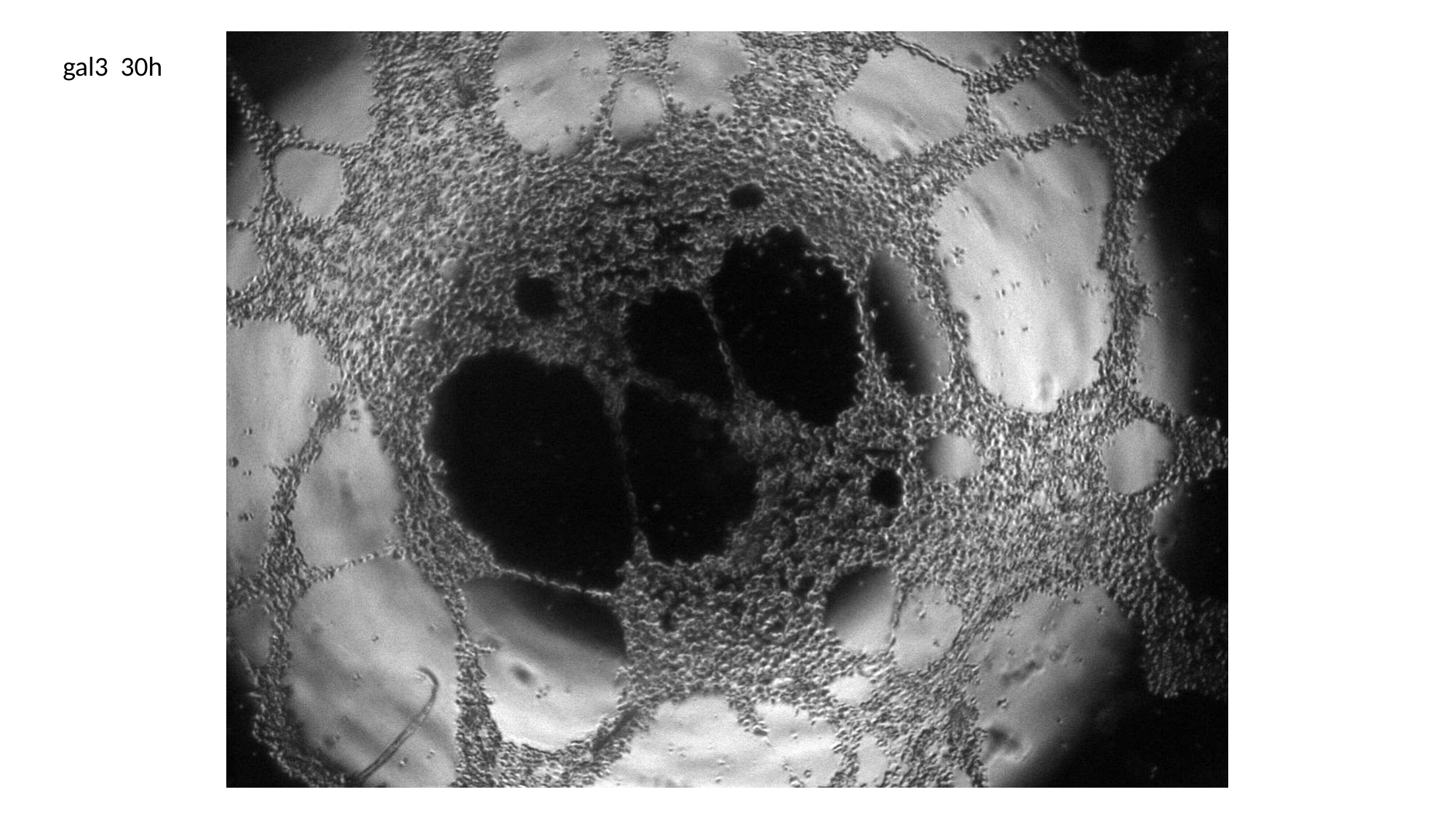

gal3 30h

## Slide 10
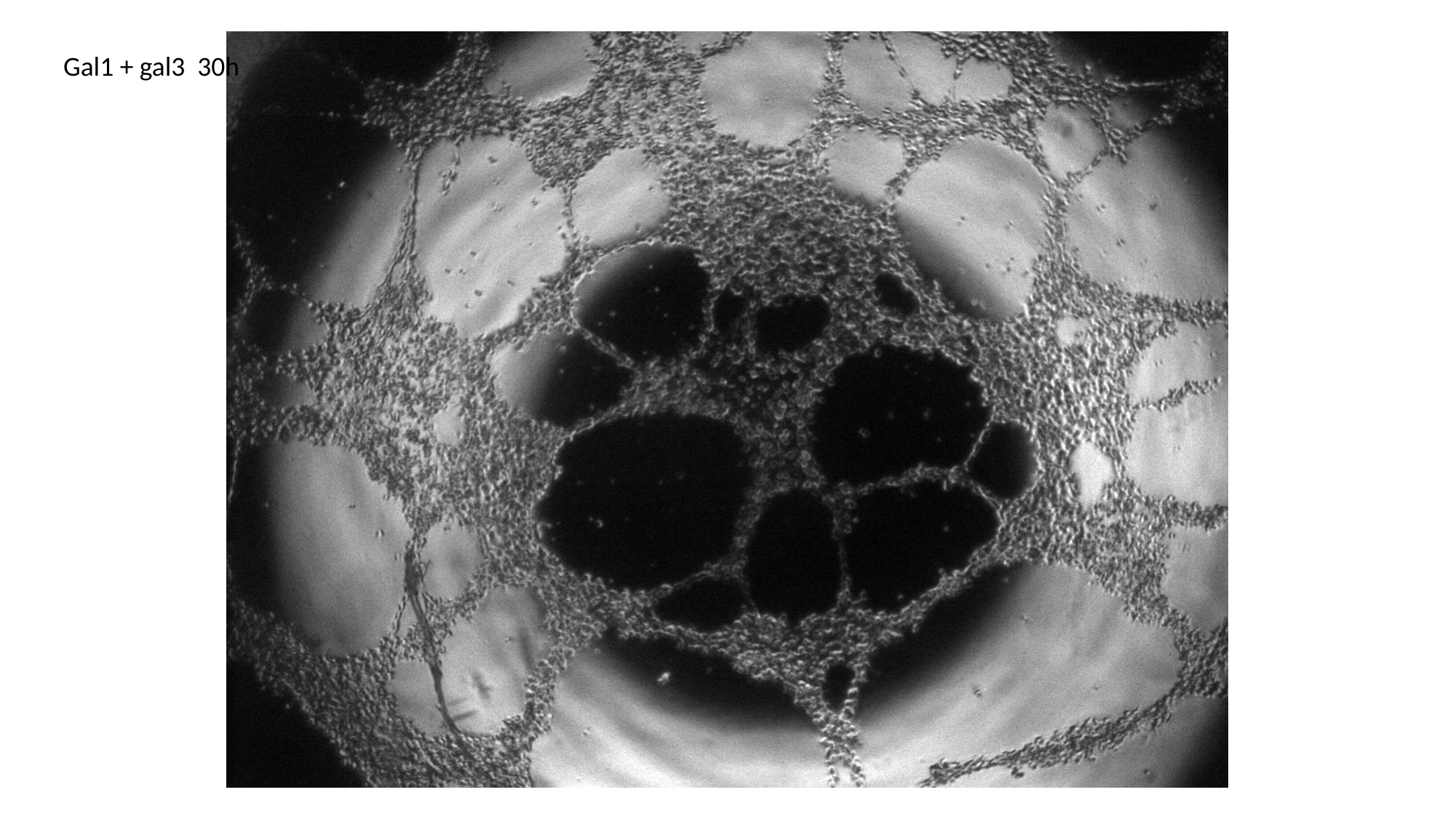

Gal1 + gal3 30h
